# Cardinality optimization in constraint-based modelling: application to human metabolism

**DOI:** 10.1093/bioinformatics/btad450

**Published:** 2023-09-11

**Authors:** Ronan M T Fleming, Hulda S Haraldsdottir, Le Hoai Minh, Phan Tu Vuong, Thomas Hankemeier, Ines Thiele

**Affiliations:** Metabolomics and Analytics Center, Leiden Academic Centre for Drug Research, Leiden University, Wassenaarseweg 76, Leiden 2333 CC, The Netherlands; Luxembourg Centre for Systems Biomedicine, University of Luxembourg, 6 avenue du Swing, Belvaux L-4362, Luxembourg; School of Medicine, National University of Ireland, University Rd, Galway H91 TK33, Ireland; Luxembourg Centre for Systems Biomedicine, University of Luxembourg, 6 avenue du Swing, Belvaux L-4362, Luxembourg; Luxembourg Centre for Systems Biomedicine, University of Luxembourg, 6 avenue du Swing, Belvaux L-4362, Luxembourg; Luxembourg Centre for Systems Biomedicine, University of Luxembourg, 6 avenue du Swing, Belvaux L-4362, Luxembourg; Mathematical Sciences School, University of Southampton, University Road, Southampton SO17 1BJ, United Kingdom; Metabolomics and Analytics Center, Leiden Academic Centre for Drug Research, Leiden University, Wassenaarseweg 76, Leiden 2333 CC, The Netherlands; School of Medicine, National University of Ireland, University Rd, Galway H91 TK33, Ireland

## Abstract

**Motivation:**

Several applications in constraint-based modelling can be mathematically formulated as cardinality optimization problems involving the minimization or maximization of the number of nonzeros in a vector. These problems include testing for stoichiometric consistency, testing for flux consistency, testing for thermodynamic flux consistency, computing sparse solutions to flux balance analysis problems and computing the minimum number of constraints to relax to render an infeasible flux balance analysis problem feasible. Such cardinality optimization problems are computationally complex, with no known polynomial time algorithms capable of returning an exact and globally optimal solution.

**Results:**

By approximating the zero-norm with nonconvex continuous functions, we reformulate a set of cardinality optimization problems in constraint-based modelling into a difference of convex functions. We implemented and numerically tested novel algorithms that approximately solve the reformulated problems using a sequence of convex programs. We applied these algorithms to various biochemical networks and demonstrate that our algorithms match or outperform existing related approaches. In particular, we illustrate the efficiency and practical utility of our algorithms for cardinality optimization problems that arise when extracting a model ready for thermodynamic flux balance analysis given a human metabolic reconstruction.

**Availability and implementation:**

Open source scripts to reproduce the results are here https://github.com/opencobra/COBRA.papers/2023_cardOpt with general purpose functions integrated within the COnstraint-Based Reconstruction and Analysis toolbox: https://github.com/opencobra/cobratoolbox.

## 1 Introduction

Systems biochemistry seeks to understand biological function in terms of a network of chemical reactions. In order to apply this approach to a particular system, one must first reconstruct the corresponding network prior to deriving a mechanistic model from it. There are various approaches to network reconstruction, depending on the type of data available and the type of computational model envisaged. Constraint-based Reconstruction and Analysis (COBRA) places strong emphasis on reconstruction of biochemical networks from complementary sources of data (Palsson 2015). Omics data is comprehensive but tends to be noisy, while biochemical data derived from reductionistic approaches is limited in scope but tends to be more precise than omics data. By combining data from these two experimental approaches one can try to ensure that a reconstructed network is comprehensive yet consistent with experimental data.

Many biochemically comprehensive genome-scale biochemical networks are available, e.g. for humans (Brunk *et al.* 2018, Robinson *et al.* 2020), yeast (Herrgård *et al.* 2008), and the human gut microbiome (Magnúsdóttir *et al.* 2017). Although reconstructions are generated based on genomic and experimental data, it is possible that incorrect or incomplete specification of biochemical reactions might still occur within any reconstruction due to errors in experimental data, human error, or incomplete experimental data. The probability of a misspecification tends to increase with the size of a reconstruction, as they tend to capture peripheral, less well studied metabolic pathways. Consequently, quality control is especially important for high-dimensional reconstructions. During the reconstruction process, quality control should be built into the reconstruction protocol in line with established best practice (Thiele and Palsson 2010), which is constantly evolving (Norsigian *et al.* 2020).

Even with quality control during the reconstruction process, it is not appropriate to assume that any reconstruction can be converted directly into a model and used to make predictions. A model must satisfy certain assumptions before it can be used to make reliable predictions. Depending on the type of model, these assumptions will be different. Each assumption should be chemically or biologically motivated and expressed in an unambiguous manner and preferably both, intuitively and mathematically. Flux balance analysis is a widely mathematical method used for studying genome-scale biochemical networks (Raman and Chandra 2009, Orth *et al.* 2010). It aims to predict steady-state reaction fluxes, where there is a balance between production and consumption of each molecular metabolites that is not exchanged across the specified boundary of the system. In this situation, one might obtain erroneous predictions if the system boundary is incorrectly specified. If a reconstruction contains one or more supposedly elementally balanced reactions, but which are actually not balanced, such reactions might lead to inadvertent leakage of a molecular metabolites from a model, in violation of mass balance. This could possibly lead to inaccurate conclusions from model predictions.

Manually testing for misspecification is impractical for genome-scale biochemical network reconstruction, with several thousand molecular metabolites and reactions. In this context, algorithmic approaches are essential to test mathematically specified modelling assumptions, isolate the subset of a reconstruction that can be used for modelling, and point towards solutions to correct the specification within a given reconstruction. Several computational tools have already been developed that can be used to test for misspecification, subject to particular assumptions in a particular modelling context. Gevorgyan *et al.* (2008) developed a variety of algorithms, and distributed software (Gevorgyan *et al.* 2011), to detect and resolve stoichiometric inconsistency, in the form of incorrectly specified mass imbalanced reactions.

Besides stoichiometric consistency, when generating a model for flux balance analysis, it is important to ensure that the model is flux consistent, i.e. each reaction can carry a nonzero steady-state flux (Vlassis *et al.* 2014). When generating a model for thermodynamic flux balance analysis (Gollub *et al.* 2021), it is important to ensure that each reaction in the model admits nonzero steady-state flux that satisfies energy conservation and the second law of thermodynamics (Qian and Beard 2005, Desouki *et al.* 2015). Such issues arises during the process of tailoring a generic genome-scale model to represent the feasible set of fluxes in a specific condition (Opdam *et al.* 2017). They are also important when generating genome-scale kinetic models, where we previously developed a stoichiometric approach to identify a form of kinetic inconsistency arising from the presence of redundant molecular metabolites (Fleming *et al.* 2016), elimination of which is necessary and sufficient for duality between unidirectional fluxes and concentrations, subject to elementary reaction kinetics.

In flux balance analysis, if no solution exists that satisfies the set of mathematical constraints specified by a model, then an industrial quality double-precision linear optimization solver will return a certificate of infeasibility, which will correctly identify an infeasible model. When dealing with a multi-scale model, e.g. with integration of metabolism and macromolecular synthesis (Thiele *et al.* 2012, Liu *et al.* 2014), a double precision solver might incorrectly return a certificate of infeasibility, when the model is actually feasible. In this scenario, reformulation to a rescaled problem (Sun *et al.* 2013) or recourse to a higher precision solver (Ma *et al.* 2017) is required.

Identification of an infeasible model is important, but it is more useful to also be able to isolate the minimal set of constraints that combine to cause the infeasibility. An irreducible infeasible set is a minimal set of constraints and variable bounds that are infeasible, but becomes feasible if any one constraint or bound is omitted (Chinneck 2008). There may also exist multiple infeasible sets of the same cardinality. A variety of different approaches exist for isolating irreducible infeasible sets in linear optimization problems (Chinneck 2008) and many industrial solvers offer the option to call such algorithms, via software for constraint-based modelling (Heirendt *et al.* 2019). However, available approaches do not exploit the sparsity of the matrices that arise in constraint-based modelling and are difficult to access for those without specialist knowledge of optimization algorithms and software.

Each of the aforementioned problems has the mathematical form of a cardinality optimization problem, a discrete optimization problem that requires minimization or maximization of the cardinality of a vector. That is, optimization of the number of nonzero components of a vector, or equivalently the zero norm of a vector. For example, in constraint-based modelling, the discreteness arises because each row or column of the stoichiometric matrix derived directly from a reconstruction must either be present or absent from a model that satisfies the aforementioned consistency conditions. Similarly, an inequality constraint can either be relaxed or not. In general, cardinality optimization subject to linear equality and inequality constraints is a computationally demanding problem, specifically, it is a nondeterministic polynomial-time hard problem (Amaldi and Kann 1998). Therefore, heuristics or approximation strategies are necessary when dealing with the high dimensional problems typical for genome-scale models. The discontinuity of the zero norm at the origin results in a nonconvex optimization problem. To circumvent the discontinuity of the zero norm, continuous approximations have been intensively studied (Le Thi *et al.* 2015). When a problem involves cardinality minimization it is referred to as sparse optimization. Efforts in this direction can be divided into two categories depending on the nature of the approximation.

In the first category, namely convex approximation, one of the best-known approaches consists of replacing the zero norm by the one norm (Tibshirani 1996). This approach leads to good performance results. Its popularity resides in its tractability and its ability to find sparse solutions, even if it is not guaranteed to attain a global minimum solution of the zero norm problem in general. However, it has been proven that, under certain sufficient conditions, a solution of a linear cardinality optimization problem can be obtained by solving a linear optimization problem with a one norm objective (Gribonval and Nielsen 2003). This is remarkable because the one norm problem is a continuous linear optimization problem that can be readily solved and may result in a solution to the original discrete cardinality optimization problem. However, the known sufficient conditions for the exact correspondence between the continuous and discrete optima are currently quite restrictive, and cannot be easily verified in general. In practice, while these methods may be efficient, the result may not be a close approximation to a true minimal cardinality solution.

In the second category, namely nonconvex approximation, the zero norm, denoted $\ell_{0}(\cdot )$ can be approximated by various continuous nonconvex functions, such as the piecewise exponential function (Bradley and Mangasarian 1998), the smoothly clipped absolute deviation (SCAD) function (Fan and Li 2001), the logarithm (Weston *et al.* 2003), the $\ell_{p+}$ norm with 0 < *P* < 1 (Fu, 1998), the $\ell_{p-}$ norm with *P *<* *0 (Rao and Kreutz-Delgado 1999), the capped-$\ell_{1}$ function (Peleg and Meir 2008), and the piecewise linear function (Le Thi *et al.* 2015). Recently, Le Thi *et al.* (2015) have shown that all of the aforementioned nonconvex approximations can be expressed as a difference of convex functions, termed a *DC function* in the mathematical literature on this topic (Pham Dinh and Le Thi 1997). This opened up the possibility to solve the aforementioned nonconvex approximation problems in a unified way using a general purpose *difference of convex function algorithm* (DCA) (Pham Dinh and Le Thi 1997). In several real applications, outside of biology, this approach has been shown to be scalable and yields better results, in terms of cardinality, than convex approximations (Le Thi *et al.* 2015).

To the best of our knowledge, there exists no report of the use of nonconvex approximations to minimize the zero norm in the context of mechanistic modelling in biology. Herein, we elaborate on several important applications of cardinality optimization that commonly appear in constraint-based modelling scenarios. We approximate them with various nonconvex approximations and we solve them with difference of convex function algorithms. The results of these and alternate approaches are compared in terms of computational efficiency and the distance of the approximation to a minimal cardinality solution for a range of genome-scale biochemical models. We place particular emphasis on real scenarios that arise while converting human metabolic reconstructions, into models suitable for flux balance analysis and thermodynamic flux balance analysis. In this context, the important interplay between algorithmically and biochemically driven resolution of inconsistencies and infeasibilities is explained.

## 2 Materials and methods

Herein, a network refers to either a reconstruction or a model derived from a reconstruction. We consider six constraint-based modelling problems involving cardinality optimization. They are (i) *stoichiometric consistency testing*: finding the maximal set of stoichiometrically consistent reactions in a network (Gevorgyan *et al.* 2008), (ii) *leak/siphon testing*: finding the minimal number of nonzero fluxes that result in a leak (or siphon) of one or more metabolites (Gevorgyan *et al.* 2008), (iii) *flux consistency testing*: finding the maximal number of reactions in a network that admit a nonzero steady-state flux (Vlassis *et al.* 2014), (iv) *thermodynamic flux consistency testing*: finding the maximal number of reactions in a network that admit a nonzero steady-state flux that satisfies energy conservation and the second law of thermodynamics (Müller and Bockmayr 2013, Desouki *et al.* 2015), (v) *sparse flux balance analysis*: finding the minimal number of nonzero fluxes that admits a particular objective (Meléndez-Hevia and Isidoro 1985), and (vi) *relaxed flux balance analysis*: finding the minimal number of bounds or mass balances to relax to render a flux balance analysis problem feasible. Of these cardinality optimization problems, our formulation of stoichiometric consistency testing and thermodynamic flux consistency testing are novel and the relaxed flux balance analysis problem has not previously been defined in the literature, so their mathematical formulation is provided below. Further details, and the mathematical formulation for the remaining problems is provided in Supplementary Section S1.1.

### 2.1 Stoichiometric consistency testing

Any stoichiometric matrix $S\in R^{m\times r}$ may be split into subsets of columns corresponding to internal and external reactions, S=[N,B], where internal reactions are stoichiometrically consistent, i.e. $\exists \ell \in R_{>0}^{m}$ such that $N^{T}\ell =0$, and external reactions are not stoichiometrically consistent, i.e. $∄\ell \in R_{>0}^{m}$ such that $B^{T}\ell =0$, as they represent net exchange of mass across the boundary of the system. Given *S* the problem is to find the largest subset that is stoichiometrically consistent. The established approach is to maximize the cardinality of a vector with dimension equal to the number of metabolites (Gevorgyan *et al.* 2008), but we take an alternate and novel approach, that minimizes the number of mass conservation constraints that need to be relaxed, subject to positive molecular mass $w\in R^{m}$ for each metabolites


(1) $$\begin{array}{cc} \underset{z,x}{\text{min}} & ||x|{|}_{0}\\ s.t. & S^{T}w+x=0,\\ & 1\leq w, \end{array}$$

where a nonzero $x\in R^{n}$ permits a relaxation of mass conservation. If $||x^{⋆}|{|}_{0}=0$ then *S* is fully stoichiometrically consistent (*S *=* N* and *B *=* *0). If $x_{j}^{⋆}=0$ for more than one reaction, then the corresponding reactions are stoichiometrically consistent. Problem 1 is a cardinality minimization problem that we approximate by a difference of convex functions and solve using a difference of convex function algorithm specialized for cardinality optimization (Supplementary Section S1.2).

In Problem (1), if $x_{j}^{⋆}\neq 0$ then the corresponding reaction is of unknown stoichiometric consistency, because one subset of reactions of unknown stoichiometric consistency may be stoichiometrically consistent in a subsequent iteration of Problem (1), if another subset of reactions of reactions of unknown stoichiometric consistency are first omitted based on certain criteria. That is, we implemented a sequential, iterative approach to identify, and subsequently omit from the network, a subset of the reactions with $x_{j}^{⋆}\neq 0$, before iterating Problem (1) again. Two criteria for reaction omission were explored. Firstly, as molecular formulae are available for most metabolites in human metabolism (Haraldsdóttir *et al.* 2014), our default approach is to omit a reaction with $x_{j}^{⋆}\neq 0$ if it was also apparently elementally imbalanced, given the chemical formulae provided. Secondly, in the case that metabolite formulae were not available, an alternate approach that avoids checking for elementally (mass) imbalanced reactions, was to omit those reactions with $x_{j}^{⋆}\neq 0$ and with the highest number of nonzero stoichiometric coefficients (lumped reactions). After each omission, Problem (1) is iterated again with a reduced stoichiometric matrix, until no smaller $||x^{⋆}|{|}_{0}$ can be obtained. After the final iteration, the reactions, and corresponding metabolites, in a reconstruction are split into those that are stoichiometrically consistent ($x_{j}^{⋆}=0$), those that are stoichiometrically inconsistent ($x_{j}^{⋆}\neq 0$ in the final iteration), and omitted reactions of unknown stoichiometric consistency ($x_{j}^{⋆}\neq 0$ in a prefinal iteration and omitted).

### 2.2 Relaxed flux balance analysis

The cardinality optimization problem, termed *relaxed flux balance analysis*, is


(2) $$\begin{array}{cc} \underset{v,r,p,q}{\min} & \lambda ||r|{|}_{0}+\alpha ||p|{|}_{0}+\alpha ||q|{|}_{0}\\ s.t. & Sv+r=b,\\ & l-p\leq v\leq u+q,\\ & p,q,r\geq 0, \end{array}$$

where $S\in R^{m\times n}$ denotes a stoichiometric matrix, $p,q\in R^{n}$ denote the relaxations of the lower and upper bounds (*l* and *u*) on reaction rates of the reaction rates vector *v*, and $r\in R^{m}$ denotes a relaxation of the mass balance constraint. Nonnegative parameters *λ* and *α* can be used to trade off between relaxation of mass balance or bound constraints, either globally, or for each individual lower bound, upper bound, or steady state constraint. Note that this problem is obliquely related to flux imbalance analysis, which starts with a feasible model and seeks to analyse the consequences of relaxing the steady state constraint for each metabolite individually and relate it to metabolite concentrations (Reznik *et al.* 2013).

### 2.3 Thermodynamic flux consistency testing

The largest set of thermodynamically flux consistent reactions is defined by the cardinality optimization problem


(3) $$\begin{array}{cc} \underset{z,w,y}{\max} & \;||z|{|}_{0}+||w|{|}_{0}\\ s.t. & \;Nz+Bw=0,\\ & l\leq \left[\begin{array}{c} z\\ w \end{array}\right]\leq u,\\ & z_{j}>0\Rightarrow N_{j}^{T}y<0,\;\forall j\in 1\ldots n,\\ & z_{j}<0\Rightarrow N_{j}^{T}y>0,\;\forall j\in 1\ldots n, \end{array}$$

where $z\in R^{n}$ is an internal reaction flux vector, $w\in R^{k}$ is an external reaction flux vector and $y\in R^{m}$ may be interpreted as a vector proportional to the chemical potentials of each metabolite (Fleming *et al.* 2012). As with flux consistency, typically all reactions in a network do not simultaneously admit a nonzero thermodynamically consistent flux so the largest set of reactions in a network that admit a thermodynamically consistent flux is the amalgamation of a set of thermodynamically flux consistent vectors. The last two constraints in Problem (3) are a relaxation of the chemical thermodynamic constraint $\text{sign}(z_{j})=-\text{sign}(N_{j}^{T}y)$ since $z_{j}=0⇎N_{j}^{T}y=0$. However, they are sufficient to identify a thermodynamically flux consistent subnetwork because any internal reaction with zero flux in all thermodynamically flux consistent vectors is considered thermodynamically flux inconsistent.

### 2.4 Cardinality optimization via a difference of convex functions

Problems (i), (ii), (iii), (v), and (vi) are each an instance of cardinality optimization over a polyhedral convex set. However, Problem (iv) requires cardinality optimization over a polyhedral nonconvex set because the set of thermodynamically feasible fluxes is nonconvex (Beard *et al.* 2004). Nevertheless, we demonstrate that each of these problems can be approximately solved by reformulation into an optimization problem, or a sequence of optimization problems, each involving a difference of convex functions (Le Thi *et al.* 2015), i.e.


(4) $$\underset{s\in R^{n}}{\min}\;\zeta (s)=\phi (s)-\varphi (s)\;$$

where $\phi (s):R^{n}\to R$ and $\varphi (s):R^{n}\to R$ are lower semi-continuous proper convex functions. As detailed in Supplementary Section S1.2, the form of the approximation is mainly determined by the choice of continuous function to approximate a step function. That is, depending on the choice of approximate step function, ϕ(s) and φ(s) may be linear or nonlinear. In our difference of convex function approach for solving (4), an outer iteration linearizes φ(s) to obtain a convex sub-problem. When ϕ is also linear, each sub-problem is a continuous linear optimization problem that may be solved using many established solvers. To summarize, in our difference of convex function approach there are two approximations. The first approximation is of the zero norm to get a difference of convex function program and the second approximation is lineariation of the second component of the difference of convex function.

### 2.5 Application to human metabolic networks

The Recon3D publication (Brunk *et al.* 2018) released a comprehensive, manually curated, genome-scale human metabolic reconstruction (ID: Recon3.01) as well a derived Recon3D model (ID: Recon3.01model) that satisfies the sufficient conditions for application of flux balance analysis, i.e. all internal reactions are stoichiometrically and flux consistent, while all external reactions are flux consistent. The Recon3D reconstruction encompasses 3297 open reading frames, 8399 metabolites, as well as 13 543 reactions distributed over nine cellular compartments: cytoplasm, lysosome, nucleus, mitochondrion, mitochondrial intermembrane space, peroxisome, extracellular space, Golgi apparatus, and endoplasmic reticulum. During quality control of Recon3D, we extensively tested our cardinality optimization algorithms as early draft versions were an amalgamation of biochemical reactions from multiple sources, which can lead to inconsistencies. The Recon3D model (ID: Recon3.01model) consists of 5385 metabolites and 10 600 reactions, derived from the Recon3D reconstruction. Besides Recon3D, a selection of other representative published human metabolic networks [Recon1.0 (Duarte *et al.* 2007), Recon2.04 (Thiele *et al.* 2013), Recon2.2 (Swainston *et al.* 2016), HMR2.0 (Mardinoglu *et al.* 2014), and Human1.0 (Robinson *et al.* 2020)] were used for numerical tests to evaluate the computational performance of difference of convex function algorithms for the aforementioned applications of cardinality optimization problems and to compare the consistency of different human metabolic reconstructions and models evaluated with different methods.

## 3 Results

### 3.1 Stoichiometric, flux, and thermodynamic flux consistency

Table 1 gives the results of testing for stoichiometric consistency, leak/siphon testing, flux consistency testing and thermodynamic flux consistency testing, computed using cardinality optimization, for seven different genome-scale human metabolic networks. A stoichiometrically consistent metabolite is one that is exclusively involved in stoichiometrically consistent reactions. A flux consistent metabolite is one that is involved in at least one flux consistent reaction, and likewise for thermodynamic flux consistency. The numbers of stoichiometrically consistent reactions and metabolites are compared with the heuristic assignment of internal and external reactions, as well as with the results of checking the elemental balance of each reaction, using chemical formulae. Table 1 reveals that all internal reactions in Recon3Dmodel are stoichiometrically consistent and flux consistent, and furthermore all external reactions are flux consistent. Recon3Dreconstruction is larger than Recon3Dmodel and does contain a subset of heuristically internal reactions that are either stoichiometrically inconsistent, flux inconsistent, or both.

**Table 1.** Comparison of human metabolic network properties.^a^

Properties	Generic human metabolic networks						
Identifier	Recon1.0	Recon2.04model	Recon2.2model	HMR2.0	Recon3.01	Recon3.01model	Human1.0
Citation	Duarte *et al.* (2007)	Thiele *et al.* (2013)	Swainston *et al.* (2016)	Mardinoglu *et al.* (2014)	Brunk *et al.* (2018)	Brunk *et al.* (2018)	Robinson *et al.* (2020)
rank [N B]	2674	4666	4945	5396	8121	5739	8161
**Reactions = Cols of [N B]**	3742	7440	7785	8181	13 543	10 600	13 520
Internal reactions = Cols of N	3310	6738	7033	7666	11 646	8791	11 795
Stoichiometrically consistent rxns.	3302	6451	7032	7655	11 345	8791	10 944
Elementally balanced reactions	3257	6231	6992	6993	10 710	8416	8412
Omitted reactions	3	30	1	7	245	0	322
Stoich. and flux consistent rxns.	2092	3646	5232	6779	8869	8791	7835
Stoich. not flux consistent rxns.	1210	2805	1800	876	2476	0	3109
Stoich. and thermo flux consistent rxns.	1884	2734	4498	5815	7546	7468	6782
Stoich. not thermo. flux consistent rxns.	1418	3717	2534	1840	3799	1323	4162
Reactions exclusive to leaks	0	0	0	0	1245	0	5
Reactions exclusive to siphons	0	0	0	0	3849	0	297
External reactions = Cols of B	432	702	752	515	1897	1809	1725
External flux consistent reactions	342	563	680	488	1816	1809	1448
**Metabolites = Rows of [N B]**	2766	5063	5324	5546	8399	5835	8438
Stoichiometrically consistent mets.	2764	5058	5317	5538	8380	5835	8408
Elementally balanced mets.	2265	3764	4465	3221	4651	3528	3026
Metabolites not stoich. consistent	2	5	7	8	19	0	30
Omitted metabolites	0	4	0	0	15	0	14
Stoich. and flux consistent mets.	1567	2289	3225	4634	5816	5835	5533
Stoich. not flux consistent mets.	1197	2769	2092	904	2564	0	2875
Stoich. and thermo flux consistent mets.	1509	1958	2984	4274	5428	5433	5108
Stoich. not thermo flux consistent mets.	1255	3100	2333	1264	2952	402	3300
Leak metabolites	0	0	0	0	1322	0	8
Siphon metabolites	0	0	0	0	3807	0	338
External mets. = Mets. exclusive to B	2	1	7	8	3	0	16

a The properties of each network may be read vertically and compared with other networks horizontally. Consider the first genome-scale human metabolic reconstruction, Recon1.0, consisting of 3742 reactions, involving 2766 metabolites. According only to heuristic assignment 432 columns of *S* correspond to external reactions and 3310 columns of *S* correspond to internal reactions. Heuristic assignment tends to underestimate the actual number of external reactions. Of the 3310 internal reactions, 3302 are stoichiometrically consistent, and the stoichiometric consistency of the remaining 8 reactions could not be confirmed so are suspected to be stoichiometrically inconsistent, especially the 3 reactions that were omitted while sequentially testing for stoichiometric consistency. Of the 3302 stoichiometrically consistent reactions only 3257 reactions are elementally balanced according to metabolite formulae pointing to the need for  an audit of the biochemical fidelity of the chemical formulae involved in the 5 mismatched reactions. Of the 3302 stoichiometrically consistent reactions, only 2092 were also flux consistent. Of 432 external reactions only 342 were also flux consistent. This subset is the part of the Recon1.0 reconstruction that can be used for prediction of mass balanced steady-state reaction rates, with no bounds on reaction rates. Tests for leaks and siphons, using the full network composed of 2766 metabolites and 3742 reactions, reveals that mass is conserved with the reaction bounds provided with Recon1.0. Here, the most important metric is the number of leak and siphon metabolites, since the corresponding cardinality for columns only shows the number of reactions exclusively involved in leakage or siphon modes, respectively. For Recon1.0, relaxing the bounds on any of the reactions that are suspect stoichiometrically inconsistent could result in leaks or siphons and unintentional violation of mass conservation in a flux balance model. Recon1.0 has two external metabolites, which have nonzero stoichiometric coefficients only in columns corresponding to external reactions. Of the 3302, and 2764, stoichiometrically consistent reactions, and metabolites, in Recon1.0, approximately 1884 reactions also admit a thermodynamically consistent nonzero flux, which corresponds to 1509 metabolites, or approximately half of the network.

As detailed in Supplementary Section S1.1.1, the stoichiometrically consistent subset of a reconstruction was identified by a sequence of iterations, where each iteration minimized the cardinality of a mass conservation relaxation vector ($x\in R^{n}$ in Problem 1) using a Difference of Convex function Cardinality Optimization (DCCO) algorithm (Le Thi *et al.* 2015). A reaction is stoichiometrically consistent if no relaxation is required ($x_{j}^{⋆}=0$). However, in all except the final iteration, a reaction is of undetermined stoichiometric consistency if relaxation is required ($x_{j}^{⋆}\neq 0$). This is because a reaction of undetermined stoichiometrically consistency may become stoichiometrically consistent if another reaction of undetermined stoichiometric consistency is omitted from the network. After each iteration, among the reactions of undetermined stoichiometric consistency, the subset of reactions that were either elementally imbalanced according to the given metabolite formulae, or the subset of reactions with the highest number of nonzero stoichiometric coefficients, were omitted and the mass conservation relaxation vector of minimial cardinality was computed again. This approach allows for computation of a larger stoichiometrically consistent subset than other published approaches (Supplementary Table S3), which underestimate the largest stoichiometrically consistent subset.

Stoichiometric consistency is invariant with respect to lower and upper bounds on reaction rates, while the results of testing for leaks, siphons, flux consistency and thermodynamic flux consistency are dependent on the lower and upper bounds on reaction rates given for each metabolic network. For example, there are no leaks or siphons in Recon1.0 (Duarte *et al.* 2007), Recon2.04 (Thiele *et al.* 2013), Recon2.2 (Swainston *et al.* 2016), HMon R2.0 (Mardinoglu *et al.* 2014), or Recon3.01model (Brunk *et al.* 2018). However, there are leaks and siphons in both Recon3.01 (Brunk *et al.* 2018) and Human1.0 (Robinson *et al.* 2020). The size of the human metabolic reconstruction, Recon3.01, and the size of the human metabolic network, Human 1.0, are almost the same. However, Recon3.01 contains 11 345 stoichiometrically consistent reactions, while Human1.0 contains 10 944 stoichiometrically consistent reactions. Also, Recon3.01 contains 8869 reactions that are both stoichiometrically and flux consistent, while Human 1.0 contains 8412 such reactions. Also, Recon3.01 contains approximately 7546 reactions that are stoichiometrically, flux and thermodynamically flux consistent, while Human 1.0 contains approximately 6782 such reactions. Recon3.01model is a model for flux balance analysis that was derived from Recon3.01. Accordingly, note that the 8791 internal reactions in Recon3.01model are all stoichiometrically and flux consistent, while all of the 1809 external reactions in Recon3.01model are flux consistent.

### 3.2 Sparse flux balance analysis

#### 3.2.1 Numerical performance

Sparse flux balance analysis (Supplementary Section S1.1.5) involves the minimization of the number of nonzero fluxes, subject to attainment of an optimal objective derived from a flux balance analysis problem $\rho^{⋆}=\max\left\{c^{T}v\;:\;Sv=b,l\leq v\leq u\right\}$. We compared the performance of sparse flux balance analysis implemented by a Difference of Convex function Cardinality Optimisation (DCCO) algorithm (Le Thi *et al.* 2015), with that of flux balance analysis (Orth *et al.* 2010) and absolute flux (one norm) minimization (Holzhütter 2004), which acts as a linear (convex) approximation of the zero norm, by a one norm. In a DCCO algorithm, the zero norm function may be approximated by variety of different continuous, nonconvex functions that approximate a step function. We performed DCCO with six continuous, nonconvex approximations to a step function (Supplementary Table S1) and then compared the results (Supplementary Table S4). DCCO with the best nonconvex approximation typically yields a smaller set of predicted active reactions than sparse optimization approximated by one-norm minimization. That is, for sparse flux balance analysis, the best nonconvex approximation outperforms a linear (convex) approximation of the zero norm.

Sparse flux balance analysis (Supplementary Problem S14) implemented with a DCCO algorithm requires the solution to a sequence of linear optimization problems. However, only two or three linear optimizations are required, even for high dimensional models, indicating the scalability of our DCCO algorithm. Moreover, we heuristically verified whether or not the set of predicted active reactions given by DCCO is minimal. One by one, we removed a reaction in the set of predicted active reactions and tested if the optimal objective value from flux balance analysis, could still be achieved. In 4 out of 6 models, the set of predicted active reactions by DCCO cannot be reduced (cf. last column in Supplementary Table S4).

#### 3.2.2 Application to Recon3D

We illustrate the utility of sparse flux balance analysis with some realistic scenarios that arise during the phase of iterative refinement of the predictive capacity of a mass balance model of metabolism. In order to mimic the requirement for energy, for maintenance of cellular integrity, many flux balance models contain a cytoplasmic adenosine triphosphate (atp[c]) hydrolysis reaction where the products are adenosine diphosphate (adp[c]) and orthophosphate (pi[c]). In Recon3D, the full corresponding reaction formula is

(5) $$\text{atp}[c]+h_{2}o\to \text{adp}[c]+\text{pi}[c]+h[c].$$

In a flux balance model, a nongrowth associated energy maintenance requirement for synthesis of adenosine triphosphate can be represented with a lower bound on reaction (5) or inclusion of this reaction within a composite biomass reaction, when cellular growth is being modelled, to represent growth associated energy requirements (Feist and Palsson 2010). In order for either of these approaches to result in a constraint on energy metabolism within the model, no stoichiometrically balanced set of internal reactions that include reaction (5) should admit isolated hydrolysis of ATP, given the reaction bounds supplied with the model. If such a set exists, sparse flux balance analysis can be used to find one such minimal cardinality set.

When all external reactions are closed, i.e. when all external reaction bounds are set to zero, then the only net flux admissible is within a stoichiometrically balanced cycle, if and only if, the bounds on each reaction in the stoichiometrically balanced cycle simultaneously admit net flux in one direction around the cycle. Net flux around a stoichiometrically balanced cycle is thermodynamically infeasible (Fleming *et al.* 2012), but steady-state mass balance constraints do not enforce thermodynamic constraints. In lieu of such constraints, the bounds on reactions can be set based on the biochemical literature to eliminate net flux around a stoichiometrically balanced cycle. In Recon3D, with all external reactions blocked (bounds are set to zero), maximizing reaction (5) while minimizing the cardinality of all internal reactions, using sparse flux balance analysis was used to find one such minimal cycle. The optimal solution involves reaction (5) in a set of nine stoichiometrically balanced reactions, with bounds that admit an arbitrary amount of isolated ATP hydrolysis (Supplementary Table S5). By further constraining the bounds to convert one reversible reaction in each such cycle to an irreversible reaction, isolated ATP hydrolysis can be eliminated, e.g. though there are important exceptions, a reaction, which hydrolyses ATP, does not generally operate in a reverse direction at biochemically realistic metabolite concentrations. This same approach can also be applied to test for the existence of a set of reactions, forming a minimal stoichiometrically balanced cycle, that admits nonzero flux through the ATP synthase reaction in the mitochondrial membrane (data not shown). Recon3Dmodel contains no set of reactions, with bounds that admit an arbitrary amount of isolated ATP hydrolysis.

#### 3.2.3 Relaxed flux balance analysis

To illustrate the utility of relaxed flux balance analysis, which uses the DCCO algorithm, we took Recon3.01model, omitted all but one biomass reaction (VMH: ‘biomass_reaction’), set a positive lower bound on the biomass reaction to require the synthesis of biomass, and close all of the external reactions. The resulting model is therefore infeasible, i.e. no steady-state flux vector satisfies the steady-state constraints and the bound constraints required by the flux balance analysis, irrespective of the objective coefficients. By solving a relaxed flux balance analysis (Problem 2), we identified a minimal set of external reaction bounds that are required to be relaxed in order to make biomass synthesis feasible. Of the 1809 external reactions, a minimal solution returned was to relax 24 lower bounds and 2 upper bounds more than the feasibility tolerance (1e−6) for a double precision optimization solver to enable biomass production (cf Supplementary Table S6). This illustrated both the utility of relaxed flux balance analysis and the dominance that biomass production has over a generic metabolic network.

## 4 Discussion

Many constraint-based reconstruction and modelling problems can be cast as cardinality optimization problems, where the objective is to minimize, or maximize, the number of nonzeros in a vector constrained to lie within a feasible set. Typically this feasible set is represents physicochemical or biochemical constraints on the set of feasible vectors. Typically this feasible set is polyhedral convex as it is constrained by a set of linear equality and inequality constraints. Cardinality optimization subject to linear equality constraints, linear inequality constraints, or both, is a nondeterministic polynomial-time hard problem in general (Amaldi and Kann 1998). It is conjectured, and widely believed in the computational complexity community, that there are no polynomial-time algorithms for such problems. As genome-scale network reconstructions continue to grow in size, approximation strategies are necessary to deal with the high dimensional cardinality optimization problems that result.

Herein, we introduce a difference of convex function approach to a selection of important cardinality optimization problems that arise in constraint-based modelling of biochemical networks. In particular, we demonstrate the quality of the cardinality approximation, the computational efficiency and the biochemical utility of this approach. Our numerical results confirm the theoretical basis for difference of convex function approaches to sparse optimization enunciated by Le Thi *et al.* (2015) and broaden the empirical evidence for the quality and computational efficiency of difference of convex function approximations to cardinality optimization problems.

A variety of different functions can be used to approximate the number of nonzeros of a vector (zero norm of a vector), which is equivalent to a sum of step functions, using a difference of convex function approach to cardinality optimization. We implemented a Difference of Convex Cardinality Optimization (DCCO) algorithm, which implemented a range of different approximate step functions. Out of six different approximations tested, overall, we find that a capped-$\ell_{1}$ approximate step function, $\psi_{\text{cap}}(t)=\min\{1,\theta |t|\}$, performs the best for the biochemical network applications considered. An added advantage of using the capped-$\ell_{1}$ approximation is that the convex problem that needs to be solved at each iteration is a linear problem. Therefore capped-$\ell_{1}$ DCCO requires the solution to a sequence of linear optimization problems, where substantial speed up can be achieved by warm starting the second and subsequent linear optimizations with the optimal solution of the previous optimization. Moreover, capped-$\ell_{1}$ DCCO offers better convergence properties than with the other approximations.

The quality of the capped-$\ell_{1}$ DCCO approximation is illustrated by our demonstration that it matches the optimal cardinality solution obtained with a mixed integer linear programming approach, when maximizing the number of conserved metabolites in a biochemical network. While MILP is consistently faster for smaller networks, Capped-$\ell_{1}$ DCCO is consistently faster for the largest models tested, that is the female and male whole body metabolic models (Thiele *et al.* 2020). In the same scenario, a linear approximation is faster, but the result is a local optimum, with cardinality below the maximum cardinality obtained with either MILP or one iteration of the capped-$\ell_{1}$ DCCO approach. As for MILP, difference of convex optimization algorithms can be used to enumerate (possibly suboptimal) solutions (Hoai An *et al.* 1998), but our implementation currently does not encompass that functionality.

We demonstrate that a difference of convex function approach to cardinality optimization is accurate and efficient for a variety of cardinality optimization problems arising in constraint-based modelling of genome-scale biochemical networks. In particular, we demonstrate that our approach is efficient for high dimensional biochemical networks confirming the broad numerical utility of our approach. We also demonstrate the biochemical utility of a difference of convex function approach to cardinality optimization by illustrative examples that arose during the process of extracting a model, suitable for flux balance analysis (Orth *et al.* 2010), from the comprehensive human metabolic reconstruction, Recon3D (Brunk *et al.* 2018). A model is suitable for flux balance analysis when all internal reactions are stoichiometrically and flux consistent, and all external reactions are flux consistent.

Due to its comprehensiveness with respect to metabolism, the Recon3D reconstruction exposes gaps in the biochemical literature. It contains content that is incompletely specified biochemically, often due to incomplete knowledge on the molecular mechanisms of certain biochemical reactions, e.g. incompletely specified reaction stoichiometry that is inconsistent with the remaining stoichiometrically consistent reactions. Stoichiometrically inconsistent reactions can be inadvertently present in draft genome-scale models, so it is important to them when extracting a model for flux balance analysis. Our new sequential cardinality optimization approach to finding the largest stoichiomerically consistent subset of a given network demonstrates that established approaches to detecting stoichiometric consistency (Gevorgyan *et al.* 2008), which are in widespread use in the community due to their incorporation into metabolic model testing portals (Lieven *et al.* 2020), underestimate the size of the largest stoichiomerically consistent subset of human metabolism. Unfortunately, this has led to erroneous conclusions regarding quality, based on a comparison of stoichiometric consistency in different human metabolic networks (Robinson *et al.* 2020).

This approach assumes that the largest set of stoichiometrically consistent reactions in a reconstruction is also consistent with biochemistry. This is more likely to be an appropriate assumption for a highly interconnected biochemical network. It is conceivable that this assumption may not be an appropriate if part of a network is poorly connected, e.g. a large set of internally consistent synthesis reactions may be inconsistent with a smaller set of consistent synthesis and degradation reactions, when the former is only connected to the rest of the network by synthesis precursors.

Recon3D contains reactions that do not admit a nonzero steady-state flux. Such flux inconsistencies are consequences of incomplete knowledge resulting in network gaps (Thiele and Palsson 2010). Associated reactions, which are not completely connected to the remainder of the metabolic network, should still be reported in the reconstruction as the reactions have generally genomic evidence, physiological evidence, or both. They represent a valuable starting point for algorithms aiming at filling such gaps (Thiele *et al.* 2014) and which thus generate novel hypotheses about missing biochemical knowledge (Reed *et al.* 2006, Thiele *et al.* 2014). Moreover, this approach also enables dynamic modelling approaches that consider the time varying function of biochemical pathways (Hoops *et al.* 2006).

Due to the high dimensionality and disparate sources of data gathered in Recon3D, the process of extracting the largest subset of the reconstruction that is suitable for a flux balance analysis provides a stiff challenge for applications of cardinality optimization in practice. Testing for stoichiometric consistency, testing for leakage or siphon of metabolites, testing for net flux consistency, testing for thermodynamic flux consistency, computing a minimal number of reactions required to be active to fulfil a certain biochemical function, and finding a minimal number of constraints to relax to make an infeasible model feasible are all cardinality optimization problems. In each case, we illustrate that a difference of convex function approach to each optimization problem does enable each problem to be efficiently solved for a variety of human metabolic networks.

**Figure 1.**
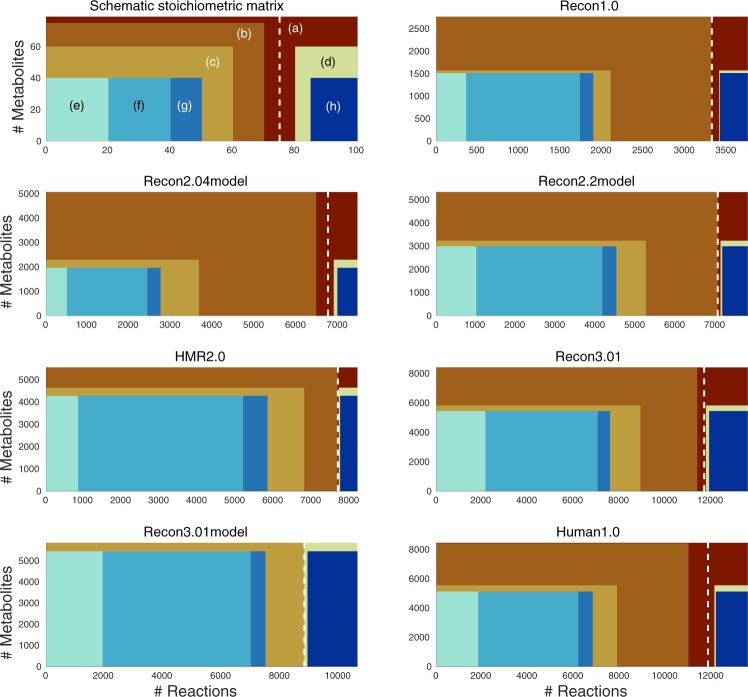
Comparison of stoichiometric, flux and thermodynamic flux consistency of human metabolic networks. A schematic stoichiometric matrix (top left) is provided for conceptual illustration. The vertical and horizontal dimensions are the number of metabolites and reactions in the network, respectively. Reactions are divided (dashed vertical line) into those heuristically internal (left) and external (right). Metabolites and reactions are split into those stoichiometric inconsistent (a) and stoichiometrically consistent (b). Of those stoichiometrically consistent, the subset if internal reactions that are also flux consistent (c), or external reactions that are flux consistent (d), using the bounds provided with the network are indicated. Of those stoichiometrically and flux consistent, using the bounds provided with the network, the approximation to the subset if internal reactions that are also thermodynamically flux consistent in both directions (e), only the forward direction (f), or only the reverse direction (g) are indicated. Of the flux consistent external reactions the approximation to the subset that are thermodynamically flux consistent (h) are indicated. The size of the stoichiometric matrix approximately doubles with each major Recon iteration, from 1.0 to 2.0 and from 2.0 to 3.0, while the size of Recon3.0 and Human1.0 are almost the same. Furthermore, in comparison with Human1.0, Recon3.01 contains more reactions that are stoichiometrically consistent, more reactions that are also flux consistent, and more reactions that are also thermodynamically flux consistent (cf Table 1 for details). Typically, only a few metabolites are exclusively involved in stoichiometrically inconsistent reactions, so the number of stoichiometrically consistent metabolites is slightly less than the number of metabolites in a network (a). Depending on the model, there are varying proportions of heuristically internal reactions that are stoichiometrically inconsistent (b, left of dashed line). In all networks, heuristically external reactions are stoichiometrically inconsistent (b, right of dashed line). Using the reaction bounds accompanying each published network, the stoichiometrically and flux consistent subset (c) and the external flux consistent subset (d) is always a strict subset of each stoichiometric matrix, except for Recon3.01model. That is, every internal reaction in Recon3.01model is stoichiometrically and flux consistent, while every external reaction is flux consistent. However, for all networks, only a subset could be confirmed to also satisfy thermodynamic flux consistency.

Comparing the stoichiometric, flux and thermodynamic flux consistency of various human metabolic networks (Fig. 1), several trends are evident. Clearly, over time, the comprehensiveness of human metabolic reconstructions has grown steadily, with the stoichiometric matrices approximately doubling in size with each release of a major Recon iteration, reflecting the results of a continual community effort. While the number of reactions that are stoichiometrically consistent does rise monotonically with each release of a human metabolic network, the fraction of reactions, and corresponding metabolites, that are stoichiometrically and flux consistent, at each model iteration does not monotonically increase. Moreover, the number of stoichiometrically, flux and thermodynamically flux consistent reactions does not montonically increase with each release of a human metabolic network. A larger reconstruction can have a lower fraction of consistent reactions in a derived model due to elimination of stoichiometrically, flux or thermodynamically flux inconsistent reactions. Due to the nonconvexity of the thermodynamically feasible steady state solution space (Beard *et al.* 2004), the predictions we have obtained of the largest thermodynamically flux consistent subset should be considered an underestimate.

A reconstruction may specify a naming scheme for heuristic categorization of reactions into internal and external sets (cf. Table1). However cardinality optimization cannot confirm that some internal reactions are stoichiometrically consistent, warranting manual curation of these reactions with suspect misspecified stoichiometry. Similarly, a reconstruction may provide elemental formulae for each metabolites and a check of elemental balance might indicate that each reaction is elementally balanced however cardinality optimization cannot confirm that some supposedly elementally balanced reactions are stoichiometrically consistent, warranting a check for misspecified stoichiometry or misspecified elemental formulae. A reconstruction may have stoichiometrically inconsistent reactions, yet the corresponding models may not have any leaking metabolites due to bounds set on the model to avoid leakage, e.g. Recon1.0. Ultimately, to avoid the possibility of occult metabolites leaks or siphons due to relaxation of bounds prior to a steady-state flux prediction, a flux balance analysis model should have no stoichiometrically inconsistent internal reactions and every reaction should be flux consistent. This is the case with Recon3Dmodel, which establishes a quality benchmark for any future flux balance model that expands beyond its current content.

## 5 Conclusions

Many constraint-based modelling problems involve optimization of the number of nonzeros in a vector subject to polyhedral convex constraints. However, no polynomial time algorithms exist for solving such cardinality optimization problems exactly. We successfully approximated a selection of high dimensional cardinality optimization problems in constraint-based modelling by solving an approximate problem involving a difference of convex functions (Le Thi *et al.* 2015). A set of corresponding difference of convex function algorithms were demonstrably efficient at solving biochemical cardinality optimization problems, including tests for stoichiometric consistency, flux consistency and thermodynamic flux consistency, as well as finding a minimal number of constraints to relax to render an infeasible flux balance problem feasible and computing metabolic pathways with minimal support. Together with our dissemination of the corresponding open source software and instructional material, we envisage that our results will theoretically and practically enable the generation of constraint-based models satisfying mathematical criteria that eliminate important modelling artefacts, which might arise with less disciplined approaches to constraint-based modelling.

## Supplementary Material

btad450_Supplementary_Data
