# Supplementary material for: Cardinality optimization in constraint-based modelling: application to human metabolism
Source: Bioinformatics. 2023 Sep 11;39(9):btad450. doi: 10.1093/bioinformatics/btad450 (PMC10495685; doi:10.1093/bioinformatics/btad450)
Supplement: btad450_Supplementary_Data [file btad450_supplementary_data.pdf]

Supplementary Information for: Cardinality optimisation in constraint-based modelling: Application to human metabolism.

1 Supplementary Methods

1.1 Applications of cardinality optimisation

We consider a biochemical network of m metabolites and r biochemical reactions. A biochemical network is mathematically represented by a stoichiometric matrix $S \in \mathbb{Z}^{m\times r}$. Each column of a stoichiometric matrix corresponds to a reaction, with negative (or positive) stoichiometric coefficients in the rows corresponding to each substrate (or product) that participates in the reaction. *External reactions* are elementally imbalanced reactions that are modelling constructs used to force a model away from thermodynamic equilibrium by exchanging one or more metabolites across the boundary of the system. A stoichiometric matrix may therefore be split horizontally into n internal and k external reactions, with stoichiometric matrices $N \in \mathbb{Z}^{m\times n}$ and $B \in \mathbb{R}^{k\times n}$, respectively, such that $S = [N, B]$, where $S = [\ ,\ ]$ denotes horizontal concatenation. Throughout, $\|\cdot\|_0$ denotes the zero norm,$\mathbf{1}$ denotes a column vector of ones,$w^\star$ denotes an optimal solution to an optimisation problem with variable w, $\mathcal{R}(S)$ denotes the range of the matrix S.

1.1.1 Stoichiometric consistency

A biochemical network is said to be *stoichiometrically consistent* if each metabolite can be assigned some positive molecular mass [1]. Equivalently, each reaction in a stoichiometrically consistent network conserves mass. That is, stoichiometric inconsistencies always result in the presence of non-conserved metabolites [2]. Besides external reactions, a network reconstruction may contain reactions with incompletely specified stoichiometry or molecules with incompletely specified chemical formulae, e.g., due to limitations in the available experimental evidence. The ideal scenario is to remove misspecified reactions from a reconstruction when generating any model subject to a mass balance constraint.

An external reaction is one that is heuristically identified by a single stoichiometric coefficient in the corresponding column of S, or an (abbreviated) reaction name matching a pattern (e.g. prefix EX_) or an external subsystem assignment. If a reaction is not external then it is denoted an internal reaction. Metabolites are external if they are exclusively involved in exchange reactions, and internal otherwise. The aforementioned assignments of external and internal reactions and metabolites is the result of a heuristic and might result in one or more errors, either due to misspecification or because the names of external reactions and external subsystems often vary between laboratories. A reaction is considered elementally balanced if the number of atoms of each element in substrates equals that of products, according to the chemical formulae specified for for each metabolites. A balanced metabolite refers to one that is exclusively involved in balanced reactions. Given chemical formulae for each metabolite, checking for elemental balancing of each reaction is more reliable than heuristic assignment. However, there is no guarantee that a set of supposedly elementally balanced reactions is stoichiometrically consistent because it is possible to erroneously specify the chemical formula for a metabolites. Therefore, method to detect stoichiometric consistency, without recourse to metabolite formulae, are required.

The problem of detecting the largest stoichiometrically consistent part of a biochemical network has previously been approached by maximising the number of metabolites that can be assigned a positive molecular mass subject to mass conservation, that is

$$\begin{array}{ll} \max\limits_{w} & \|w\|_0 \\ \text{s.t.} & S^T w = 0, \\ & w \geq 0, \end{array} \tag{6}$$

where $w \in \mathbb{R}^m$ is proportional to the molecular mass of each metabolites, and the optimal solution $w^\star$ is referred to as a maximal conservation vector. The equality constraint enforces mass conservation (for positive molecular mass). If $\|w^\star\|_0 = m$ then S is stoichiometrically consistent ($S = N$ and $B = 0$), otherwise it is not. If $w_i^\star = 0$ then the mass of the corresponding metabolites is not conserved by the reactions in the network. Mixed-integer linear [1] and linear approximations [3] to Problem (6) have previously been explored.

Gevorgyan et al. [1] reformulated (6) as a mixed-integer linear problem (MILP)

$$\begin{array}{ll} \max\limits_{w,t} & \mathbf{1}^T t \\ \text{s.t.} & S^T w = 0 \\ & w \geq t \\ & t \in \{0,1\}^m \end{array} \tag{7}$$

Since there is no upper bound for w, the number of non-zero components of w will reach the possible maximum, i.e., globally solving (7) would give the largest possible stoichiometrically consistent part of the given biochemical

network. However, there are no efficient algorithms for solving Problem (7) that can also provide a guarantee of global optimality. Moreover, approximately solving Problem (7) with an MILP solver can be time-consuming for very large-scale models.

Fleming et al. [3] approximated (6) by the linear optimisation problem

$$\begin{array}{ll} \max\limits_{w,t} & \mathbf{1}^T t \\ \text{s.t.} & S^T w = 0 \\ & w \geq t \\ & 0 \leq t \leq \mathbf{1}\alpha \\ & 0 \leq w \leq \mathbf{1}\beta \end{array} \tag{8}$$

where $t \in \mathbb{R}^m$. Since the constraint $w \geq 0$ can not be enforced numerically with floating point, it is replaced by $w \geq \mathbf{1}\alpha$ where α is an arbitrary positive number. The scalars α, β are proportional to the smallest molecular mass considered non-zero and the largest molecular mass allowed. Our initial aim was to solve the Problem (6) by using non-convex approximations. It is clear that (6) is a special case of the main problem (21), in which $y = w$ and x,z are omitted. However, in practical situations, we observed that it often occurs that one can obtain a larger value for $\|w^\star\|_0$ if a stoichiometrically inconsistent reaction is first removed from the network. Even if that removal of that reaction causes a reduction in the number of metabolites in the network, it is still possible for $\|w^\star\|_0$ to be larger than before. A typical example is when one removes an imbalanced reaction involving a large number of metabolites that otherwise participate in balanced reactions. This is the rationale for the new method for stoichiometric consistency testing described in Section 2.1.

1.1.2 Leaks and siphons

In standard notation, flux balance analysis is the linear optimisation

$$\begin{array}{ll} \min\limits_{v} & \rho(v) \coloneqq c^T v \\ \text{s.t.} & Sv = b, \\ & l \leq v \leq u, \end{array} \tag{9}$$

where $c \in \mathbb{R}^r$ is a parameter vector that linearly combines one or more reaction fluxes to form what is termed the objective function and where a $b_i < 0$, or $b_i > 0$, represents some fixed output, or input, of metabolites i. If $b = 0$, a metabolites *may* be produced from nothing (leak metabolites) or consumed for nothing (siphon metabolites) in violation of mass conservation, if all reactions are reversible and S is stoichiometrically inconsistent [1]. However, even with a stoichiometrically inconsistent S, mass conservation *may still not be* violated if the lower and upper bounds on reaction rates, $l, u \in \mathbb{R}^n$ prevent it. For instance, the reactions $A + B \rightleftharpoons C$ and $C \rightleftharpoons A$ are stoichiometrically inconsistent because it is impossible to assign a positive molecular mass to all metabolites whilst ensuring that each reaction conserves mass. However, if the bounds are such that both reactions are unidirectional in the forward direction, $A + B \rightarrow C$ and$C \rightarrow A$, then leakage of B is no longer possible and so mass is conserved.

Testing for leaks or siphons has been widely used in genome-scale metabolic modelling, prior to distribution of a model for prediction of mass conserved steady-states [4]. One can identify all leaks in a network with the cardinality optimisation problem

$$\begin{array}{ll} \min\limits_{v,y} & \|y\|_0 \\ \text{s.t.} & Sv - y = 0, \\ & l \leq v \leq u, \\ & 0 \leq y, \end{array} \tag{10}$$

where$y_i^\star > 0$ indicates that the corresponding metabolites is a leak and can be produced from nothing. To test for siphons, replace the equality constraint in (10) with $Sv + y = 0$. This approach is useful when double checking stoichiometric consistency, with the bounds for all reactions that are known to be stoichiometrically inconsistent set to zero.

A minimal number of reactions required to be active and result in a leak of the metabolites subset Ω can be found using the cardinality optimisation problem

$$\begin{array}{ll} \min\limits_{v,y} & \|v\|_0 \\ \text{s.t.} & Sv - y = 0, \\ & l \leq v \leq u, \\ & 0 \leq y, \\ & 1 \leq y_i, \ i \in \Omega \end{array} \tag{11}$$

with $v^{\star}$ referred to as a minimal leakage mode [1]. A minimal siphon mode would be obtained if the equality constraint was replaced with $Sv+y=0$. Problem 10 is a cardinality minimisation problem that we approximate by a difference of convex functions and solve using a difference of convex function algorithm specialised for cardinality optimisation (Supplementary Section 1.2).

Similarly, a minimal number of reactions contributing to leakage of a minimal number of metabolites can be found using the cardinality optimisation problem

$$\begin{array}{ll} \min\limits_{v,y} & \|v\|_0 + \|y\|_0 \\ \text{s.t.} & Sv - y = 0, \\ & l \leq v \leq u, \\ & 1 \leq v_j, \ j \in \Omega \\ & 0 \leq y, \end{array} \tag{12}$$

where the additional inequality enforces the rate of a subset of reactions (Ω) to be active. Considering Problem (1, main text), the utility of Problems (11) and (12) is in the identification of reactions within the suspect stoichiometrically inconsistent set, to remove prior to reiteration of Problem (1, main text) with a reduced network. This requires manual curation of the results of Problems (11) and (12), where the advantage is that they often narrow down significantly the search for stoichiometrically inconsistent reactions, especially in the context of genome-scale networks.

1.1.3 Flux consistency

If one assumes that all molecules are at steady-state, the corresponding computational model should be *flux consistent*, meaning that each net reaction of the network has a non-zero flux in at least one feasible steady-state net flux vector. For example, consider the set of reactions

$$\emptyset \rightleftharpoons D \rightleftharpoons G \rightleftharpoons \emptyset, \qquad D \rightleftharpoons H. \tag{13}$$

In this set, the reaction $D \rightleftharpoons H$ is flux inconsistent, as any non-zero net flux is inconsistent with the assumption that the concentration of H should be time invariant. Inclusion of flux inconsistent reactions, like $D \rightleftharpoons H$, in a dynamic model would be perfectly reasonable, but we omit such reactions because the focus of this paper is on modelling of steady-states.

The largest set of flux consistent reactions in a network can be obtained by solving the following cardinality optimisation problem

$$\begin{array}{rl} \max\limits_{V \in \mathbb{R}^{r \times p}} & \mathbf{1}^T \cdot [\![V]\!] \\ \text{s.t.} & SV = 0, \\ & l \cdot \mathbf{1} \leq V \leq u \cdot \mathbf{1}, \end{array} \tag{14}$$

where $V \in \mathbb{R}^{r \times p}$ denotes a matrix of $p \leq r - \text{rank}(S)$ horizontally concatenated flux vectors and $[\![\cdot]\!] : \mathbb{R}^{r \times p} \rightarrow \mathbb{R}^{r \times p}$ denotes an entry-wise application of a step function (cf. Supplementary Figure 1), with $[\![V_{i,j}]\!] = \varsigma(V_{i,j}) = 1$ if $V_{i,j} \neq 0$ and $\varsigma(V_{i,j}) = 0$ otherwise.

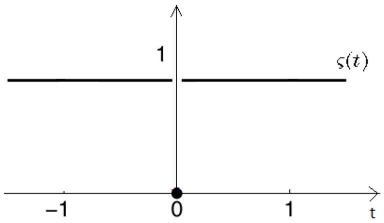

Figure 1: Step function ς.

For most biochemical networks, all reactions can not be simultaneously active in one flux mode, making flux consistency testing a form of matrix cardinality maximisation problem, where the maximum number of flux modes is bounded by the column rank deficiency of the stoichiometric matrix. We say a reaction, corresponding to the jth column of S, is flux consistent if the corresponding row of an optimal solution $V^{\star}$ to Problem (14) contains at least one non-zero entry.

Previously Vlassis et al [5] used a non-convex approximation to a step function to approximate maximisation of the cardinality of a steady state flux vector. This approach took advantage of the fact that the cardinality function is quasiconvex on the positive orthant, which suited irreversible reactions but requires a heuristic

approach to flip the direction of reversible reactions. In general the cardinality function is not quasiconvex. Problem 14 is a cardinality maximisation problem that can be approximated by a difference of convex functions and solved using a difference of convex function algorithm specialised for cardinality optimisation (Supplementary Section 1.2). Following Vlassis et al [5], each approximate solution to Problem (15) is obtained by using linear optimisation to maximise the approximate step function, $\psi_{cap}(t) = \min\{1, \theta|t|\}$. Note that this step function may be represented as a difference between two convex functions (cf Table1). This algorithm approximately solves Problem (14) by approximately solving a sequence of k cardinality maximisation problems, of the form

$$\begin{aligned} \max_{v} \ & \|v\|_0 \\ \text{s.t. } & Sv = 0, \\ & l \leq v \leq u. \end{aligned} \tag{15}$$

The result is a matrix $V \in \mathbb{R}^{n \times k}$ of $k \leq n - r$ flux modes such that $SV = 0$ and $l \cdot \mathbf{1} \leq V \leq u \cdot \mathbf{1}$. The set of flux consistent metabolites is obtained by identification of each metabolite that participates in a flux consistent reaction. The remaining set of metabolites, i.e., flux inconsistent metabolites, but also referred to as dead-end metabolites in the literature [4], correspond to the metabolites that are exclusively involved in flux inconsistent reactions.

1.1.4 Thermodynamic flux consistency

Any stoichiometric matrix S may be split into one subset of columns corresponding to internal and external reactions, $S = [N, B]$, where internal reactions are stoichiometrically consistent, that is $\exists \ell \in \mathbb{R}^m_{>0}$ such that $N^T\ell = 0$, and external reactions are not stoichiometrically consistent, that is $\nexists \ell \in \mathbb{R}^m_{>0}$ such that $B^T\ell = 0$, as they represent net exchange of mass across the boundary of the system. Previously, Desouki et al. [6] introduced the CycleFreeFlux algorithm, which reduces a given internal reaction flux vector z' to its thermodynamically feasible part $z^\star$, obtained at the optimum of a linear optimisation problem of the form

$$\begin{aligned} \min_{z,w} \quad & \|z\|_1 \\ \text{s.t.} \quad & Nz + Bw = 0, \\ & c^T \cdot w = c^T \cdot w', \\ & w = w' \\ & 0 \leq z_j \leq z'_j \ \forall j : z'_j \geq 0, \\ & 0 \leq z_j \leq z'_j \ \forall j : z'_j < 0, \end{aligned} \tag{16}$$

where $z \in \mathbb{R}^n$ and $w \in \mathbb{R}^k$ are internal and external reaction fluxes, respectively. Here $\|z\|_1$denotes the one-norm of internal reaction fluxes and $c \in \mathbb{R}^k$ is a vector of objective coefficients, restricted only to external reaction fluxes (cf Desouki et al. [6], where $c \in \mathbb{R}^r$). The last three sets of constraints require that external reaction fluxes remain unchanged and that the direction of internal reaction flux does not change, but are allowed to become equal to zero in magnitude.

Inspired by Desouki et al. [6], we observed that a thermodynamically feasible flux may be computed by a single linear optimisation problem

$$\begin{aligned} \min_{z,w} \quad & \|z\|_1 + c^T \cdot w \\ \text{s.t.} \quad & Nz + Bw = 0 \ \ : y \\ & \overline{z} \leq z \leq \underline{z} \ \ : s \\ & \overline{w} \leq w \leq \underline{w} \ \ : t \end{aligned} \tag{17}$$

where $\underline{z}$ and $\overline{z}$ denote lower and upper bounds on internal reaction fluxes, with the constraint that $\underline{z} \in \{0, -\infty\}^n$ and $\overline{z} \in \{0, \infty\}^n$, while $\underline{w} \in \mathbb{R}^k$ and $\overline{w} \in \mathbb{R}^k$ denote lower and upper bounds on external reaction fluxes, respectively. The vectors $y \in \mathbb{R}^m$, $s \in \mathbb{R}^m$ and $t \in \mathbb{R}^m$, are dual variables to the steady state constraint, bounds on internal reaction rates and bounds on external reaction rates, respectively.

The optimality conditions of Problem 17 are

$$\begin{aligned} Nz^\star + Bw^\star &= 0 \\ \nabla \|z^\star\|_1 = \text{sign}(z^\star) &= N^T y^\star + s^\star \\ c &= B^T y^\star + t^\star \end{aligned}$$

where $-y^\star$ may interpreted as a vector proportional to the chemical potentials of each metabolite and $-N_j^T y^\star$ is proportional to the change of chemical potential for reaction j.When $\underline{z} \in \{0, -\infty\}^n$ and $\overline{z} \in \{0, \infty\}^n$ then the optimal dual variable $s_j^\star$ to the inequality constraints on internal reaction j, is non-zero if and only if $z_j^\star$ is zero, that is $z_j^\star = 0 \iff s_j^\star \neq 0$ and $z_j^\star \neq 0 \iff s_j^\star = 0$. Therefore $z_j^\star \neq 0 \iff \text{sign}(z_j^\star) = N_j^T y^\star$, which enforces

energy conservation and the second law of thermodynamics on the optimal vector of nonzero internal reaction fluxes [7]. However, when $z_j = 0$, this is a relaxation of the thermodynamic constraint $\text{sign}(z_j) = -\text{sign}(N_j^T y)$, since $z_j^\star = 0 \iff \text{sign}(z_j^\star) = N_j^T y^\star + s_j^\star = 0$, so $z_j^\star = 0 \iff N_j^T y^\star = -s_j^\star$ and therefore $z_j = 0 \not\Leftrightarrow N_j^T y = 0$. Biochemically, one may interpret this relaxation as saying that a zero internal reaction flux does not imply a zero change in chemical potential. For example, a nonzero change in chemical potential may still be consistent with zero net flux when an enzyme is absent for the corresponding reaction. To summarise, herein we define thermodynamic consistency as the requirement that any nonzero net flux be driven by a change in chemical potential for the corresponding reaction, that is

$$\begin{aligned} z_j^\star > 0 \quad &\Rightarrow \quad N_j^T y^\star < 0, \\ z_j^\star < 0 \quad &\Rightarrow \quad N_j^T y^\star > 0, \end{aligned}$$

However, a reactions must admit a non-zero flux that is thermodynamically consistent to be deemed thermodynamically flux consistent, so we omit reactions where zero net flux is the only thermodynamically consistent solution obtained.

Problem 17 may be used to compute a single flux vector that is thermodynamically feasible. One must compute multiple thermodynamically feasible net flux vectors in order to approximate the largest thermodynamically consistent subset of a given flux consistent model, because different combinations of reactions can be active in thermodynamically feasible fluxes with disjoint sparsity patterns. Inspired by the use of randomly weighted objectives to efficiently explore the set of flux consistent reactions [8], by default, we employ a similar strategy to bias toward thermodynamically feasible flux vectors that have non-zero flux in random subsets of reactions. A greedy sequence of optimisation problems is then used to iteratively increase the number of reactions in the thermodynamically consistent subset. At each iteration the following cardinality optimisation problem is solved

$$\begin{array}{ll} \min\limits_{z,w,p,q} & g^T \|z\|_0 + \beta 1^T (p+q) \\ \text{s.t.} & Nz + Bw = 0 \\ & z - p + q = 0 \\ & \underline{z} \le z \le \overline{z} \\ & \underline{w} \le w \le \overline{w} \\ & 0 \le p \\ & 0 \le q \end{array} \tag{18}$$

where $g^T \|z\|_0$ denotes the weighted zero-norm of internal reaction fluxes, where the zero-norm for each reaction is weighted individually, that is

$$g^T \|z\|_0 \coloneqq \sum_{j=1}^{n} g_j^T \|z_j\|_0 \,.$$

Here $g \in \mathbb{R}^n$ is standard random vector generated from the zero-mean normal distribution in the open interval $(-0.5, 0.5)$ (the random part), except $g_j = 0$ if the jth reaction has already been identified as part of the thermodynamically consistent set (the greedy part). Minimisation of the one-norm of internal net reaction flux, to promote thermodynamic feasibility [6], is achieved by constraining net flux to be equal to the difference between two non-negative vectors, that is $z = p - q$ and minimising the sum of forward net reaction flux $p \in \mathbb{R}^n_{\ge 0}$ and reverse net reaction flux $q \in \mathbb{R}^n_{\ge 0}$. Bounds on z and w are the same as in Problem 17. Each iteration of Problem 18 is efficiently solved using a sequence of optimisation problems, each involving a difference of convex functions [9]. Depending on the signs of non-zero z_j, one may identify whether a reaction admits thermodynamically consistent flux in either the forward direction, reverse direction, or both.

Given a solution Problem 18, thermodynamic consistency can also be checked using a Problem (16). The scalar weight $\beta > 0$ is chosen to trade off between cardinality optimisation of internal reaction fluxes, to explore the flux consistent subset of the model, and minimising the one norm of the sum of forward and reverse reaction rates, to promote thermodynamic consistency. By default, $\beta = 0.1$, which computational experiments determined to be approximately optimal for genome-scale models tested. If β is too small, too few of the reaction fluxes are thermodynamically feasible, while if β is too large, exploration of the thermodynamically consistent set is impeded.

Any internal reaction with a *forcing bound*, that is a positive lower bound ($\underline{z_j} > 0$), or a negative upper bound ($\overline{z_j} < 0$), can indirectly force steady state flux in other internal reactions, which may or may not be thermodynamically consistent, depending on the bounds on external reactions. If the direction of the bounds on external reactions is consistent, or inconsistent, with the direction of the bounds of the forced internal reaction, the directly and indirectly forced internal reactions admit, or do not admit, a thermodynamically consistent steady state flux, respectively. The difference between these alternatives can be identified by implementing the forcing bounds, by further constraining in Problem (18), allowing relaxation to zero of each bound that

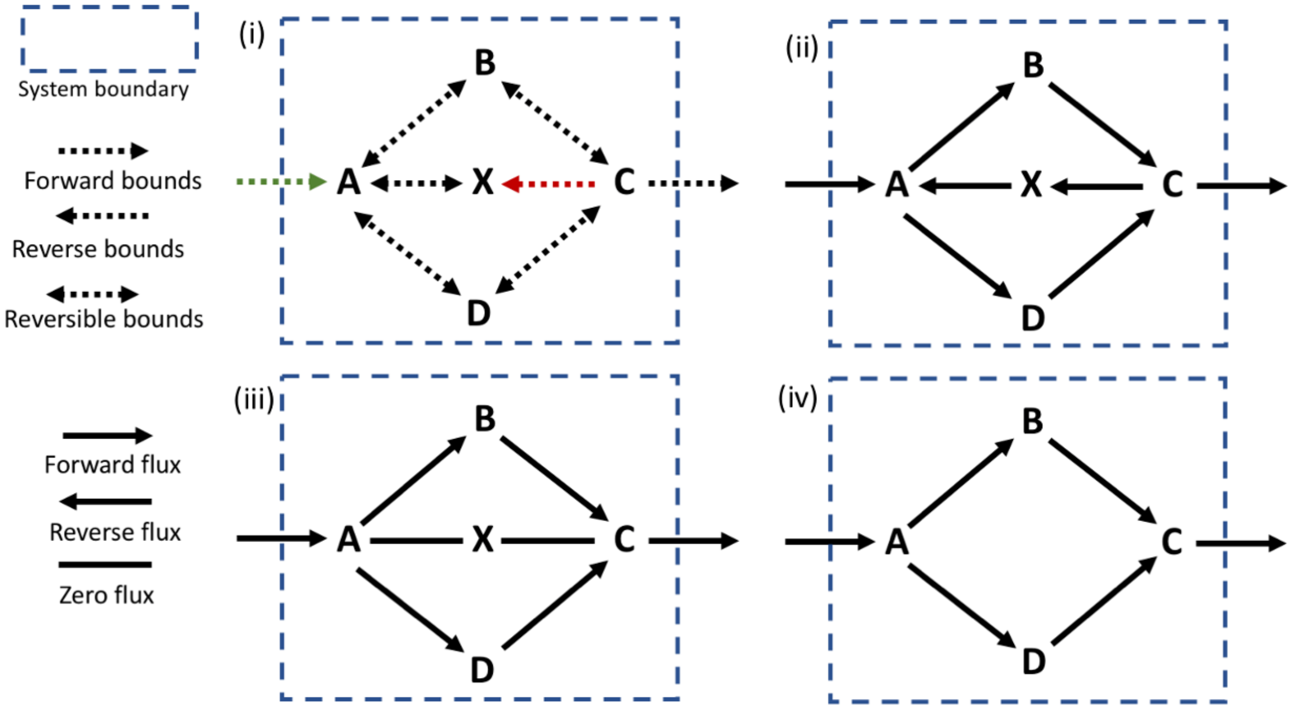

Figure 2: **Thermodynamic flux consistency of a toy network**. (i) Network where the lower bound on external reaction $\emptyset \to A$ (green) and internal reaction C→X (red) force flux through parts of the network. (ii) Network with all reactions flux consistent. Note that this is only possible with thermodynamically inconsistent cyclic flux around a stoichiometrically balanced cycle, e.g., $A \to B \to C \to X \to A$. (iii) All internal reactions are thermodynamically consistent, but reactions C→X and X→A have zero flux, so are not thermodynamically flux consistent. (iv) A fully thermodynamically flux consistent subnetwork. Note omission of reaction C→X and X→A, which are thermodynamically flux inconsistent with the bounds given for external reaction $\emptyset \to A$ (green).

forces internal reaction flux, promoting non-zero flux in such reactions in Problem (16), then observing if thermodynamically feasible net flux is possible in the original direction, with magnitude equal or greater to that required by the forcing bound. The toy network illustrated in Figure 2 illustrates a key difference between flux consistency and thermodynamic flux consistency of steady state fluxes.

1.1.5 Sparse flux balance analysis

A typical application of flux balance analysis is to predict an optimal non-equilibrium steady-state flux vector that optimises a linear objective function, such biomass production rate, subject to bounds on certain reaction rates. It is a cardinality minimisation problem to predict the minimal number of active reactions required to carry out a particular set of biochemical transformations[10], consistent with an optimal objective derived from the result of a standard flux balance analysis problem. However, for many biochemical systems it is not clear what the correct objective is. An alternative approach is to tighten the constraints on steady-state fluxes, either by eliminating flux through reactions whose gene products are absent from the cell type being modelled or by tightening the bounds on active reactions, then minimise the number of reactions required to be active to satisfy those constraints. In each case, the corresponding problem is a cardinality minimisation problem, that we term *sparse flux balance analysis*, is

$$\begin{array}{ll} \min\limits_{v} & \|v\|_0 \\ \text{s.t.} & Sv = b \\ & l \leq v \leq u \\ & c^T v = \rho^\star \end{array} \tag{19}$$

where the last constraint is optional and represents the requirement to satisfy an optimal objective value $\rho^\star$ derived from any solution to Problem (9). The optimal flux vector can be considered a steady-state biochemical pathway with minimal support, subject to the bounds on reaction rates and satisfaction of the optimal objective. Note also that Problem (19) is is a variant of Problem (12), to compute the minimal number of reactions

contributing to leakage of a minimal number of metabolites. In both cases, we require minimisation of the number of active reactions, if one considers y in Problem (12) as a unidirectional external reaction.

1.1.6 Relaxed flux balance analysis

Every solution to Problem (9) must satisfy $Sv = b$ and $l \leq v \leq u$, independent of any objective chosen to optimise over the set of constraints. It may occur that these constraints are not all simultaneously feasible, i.e., the system of inequalities is infeasible. This situation might be caused by an incorrectly specified reaction bound or the absence of a reaction from the stoichiometric matrix, such that a non-zero $b \notin \mathcal{R}(S)$. To resolve the infeasibility, we consider a cardinality optimisation problem that seeks to minimise the number of bounds to relax, the number of fixed outputs to relax, the number of fixed inputs to relax, or a combination of all three, in order to render the problem feasible.

The cardinality optimisation problem, that we term *relaxed flux balance analysis,* is

$$\begin{array}{ll} \min\limits_{v,r,p,q} & \lambda\|r\|_0 + \alpha\|p\|_0 + \alpha\|q\|_0 \\ \text{s.t.} & Sv + r = b \\ & l - p \leq v \leq u + q \\ & p, q, r \geq 0 \end{array} \tag{20}$$

where $p, q \in \mathbb{R}^n$ denote the relaxations of the lower and upper bounds on reaction rates of the reaction rates vector v, and where $r \in \mathbb{R}^m$ denotes a relaxation of the mass balance constraint. Non-negative scalar parameters λ and α can be used to trade off between relaxation of mass balance or bound constraints.

A non-negative vector parameterλ can be used to prioritise relaxation of one mass balance constraint over another, e.g, to avoid relaxation of a mass balance constraint on a metabolite that is not desired to be exchanged across the boundary of the system. A non-negative vector parameterα may be used to prioritise relaxation of bounds on some reactions rather than others, e.g., relaxation of bounds on exchange reactions rather than internal reactions. Note that if $b = 0$, and $\text{rank}(S) < n$ there always exists a non-zero solution to $Sv = 0$ if $l = -\infty$ and $u = \infty$, that is, if $b = 0$ any infeasibility must come from the bounds and we can fix $r = 0$ leaving only p and q for relaxation of bounds. The optimal choice of parameters depends heavily on the biochemical context. A relaxation of the minimum number of constraints is desirable because ideally one should be able to biochemically justify each bound to relax, or each additional metabolites to be exchanged across the boundary of the system, by referencing the experimental literature. This task is roughly proportional to the the number of constraints proposed to be relaxed.

1.2 Cardinality optimisation and differences of convex functions

Each of the aforementioned cardinality optimisation problems is an instance, or a sequence, of the following general cardinality optimisation problem

$$\begin{array}{ll} \min\limits_{x,y,z} & c^T \begin{bmatrix} x \\ y \\ z \end{bmatrix} + \lambda\|x\|_0 - \delta\|y\|_0 \\ \text{s.t.} & A \begin{bmatrix} x \\ y \\ z \end{bmatrix} \leq b, \\ & l \leq \begin{bmatrix} x \\ y \\ z \end{bmatrix} \leq u, \\ & x \in \mathbb{R}^p,\ y \in \mathbb{R}^q,\ z \in \mathbb{R}^r, \end{array} \tag{21}$$

where $c \in \mathbb{R}^{p+q+r}$ is a parameter vector in a linear objective function, the second term in the objective minimises the cardinality of the variable vector x, while the third term maximises the cardinality of the variable vector y. The data in $A \in \mathbb{R}^{s\times(p+q+r)}$ and $b \in \mathbb{R}^s$ implement linear equality or inequality constraints, supplemented by box constraints on the variables, $l, u \in \mathbb{R}^{p+q+r}$. The parameters $\lambda, \delta > 0$ can be used to trade-off between minimisation of the cardinality of x and maximisation of the cardinality of y.

1.2.1 Difference of convex functions

Consider the following general optimisation problem involving a difference of convex functions

$$\min_{s\in\mathbb{R}^n} \zeta(s) := \phi(s) - \varphi(s) \tag{22}$$

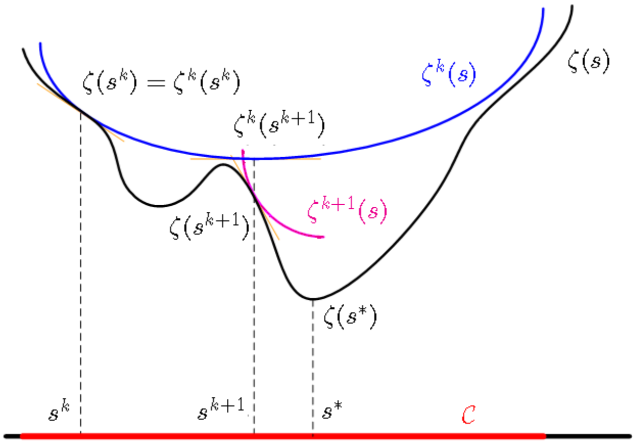

Figure 3: **Difference of convex function algorithm.** This algorithm can minimise any function $\zeta(s) : \mathbb{R}^n \to \mathbb{R}$ that can be expressed as a difference of convex functions $\zeta(s) := \phi(s) - \varphi(s)$, over a convex set $\mathcal{C}$. The key idea is that, at each iteration one linearly approximates the non-convex part of $\zeta(s)$ and then minimises the resulting convex function. Suppose that at iteration k, the current solution is s^k. One replaces $-\varphi(s)$ by its affine majorisation to obtain the convex function $\zeta^k(s) := \phi(s) - \varphi(s^k) - (s - s^k)^T \bar{s}^k$ where$\bar{s}^k$ belongs to the subdifferential of φ ats^k. Note that $\zeta(s) \leq \zeta^k(s)$ and $\zeta(s^k) = \zeta^k(s^k)$. Since $\zeta^k(s)$ is a convex function, one can globally solve the convex problem $\min_{s \in C} \zeta^k(s)$ to obtain the solutions^{k+1}. Then one reiterates the previous steps until convergence of the sequence s^k toward a minimal $\zeta(s^k)$.

where $\phi(s) : \mathbb{R}^n \to \mathbb{R}$ and$\varphi(s)$ are lower semi-continuous proper convex functions. Note that while $\varphi(s)$ is convex, the part $-\varphi(s)$ of $\zeta(s)$ is not. If s belongs to an arbitrary convex set $\mathcal{C} \subset \mathbb{R}^n$, the problem

$$\min_{s \in C} \zeta(s) = \phi(s) - \varphi(s)$$

can be equivalently reformulated in the general form of Problem (4, main text) [11, 12]. A given difference of convex function has infinitely many decompositions into a difference of two convex functions (DC functions). Therefore, there is the potential for as many different difference of convex function algorithms (DCA) as there are decompositions. The decomposition used in practice plays a critical role in determining the speed of convergence, robustness, and quality of solutions. This flexibility is of particular interest from a theoretical and an algorithmic point of view. For a complete study of DC functions and DCA, the reader is referred to [11, 12] and the references therein. The overall approach of a difference of convex function algorithm [11, 12] is geometrically illustrated in Figure 3 and generic algorithm is specified as follows:

Difference of Convex Function Algorithm (DCA):

1. Let s^0 be any initial point andϵ be a sufficiently small tolerance. Set $k = 0$.
2. Compute$\tilde{s}^k$ which belongs to the subdifferential of φ at s^k:$\tilde{s}^k \in \partial\varphi(s^k)$.
3. Compute s^{k+1} by solving the convex optimisation problem

$$(\mathcal{P}_k) \ \min_{s} \zeta^k(s) := \phi(s) - \varphi(s^k) - (s - s^k)^T \tilde{s}^k.$$

4. If $\|s^{k+1} - s^k\| < \epsilon$ or $\|\zeta(s^{k+1}) - \zeta(s^k)\| < \epsilon$ then STOP and RETURN s^{k+1}, otherwise set $s^k = s^{k+1}$, set $k = k + 1$, and go to Step 2.

1.2.2 Difference of convex function approximation of cardinality optimisation

Consider the step function $\varsigma(t) : \mathbb{R} \to \mathbb{R}$ where $\varsigma(t) = 1$ if $t \neq 0$ and $\varsigma(t) = 0$ otherwise, illustrated in Figure 1. The zero norm of a vector $s\in \mathbb{R}^n$ is equivalent to a separable sum of step functions, $\|s\|_0 = \sum_{i=1}^{n} \varsigma(s_i)$. Let $\psi_\theta(t) : \mathbb{R} \to \mathbb{R}$ be an approximation of the step function $\varsigma(t)$. $\psi_\theta(t)$ depends on a *step parameter* $\theta \in \mathbb{R}$ and satisfies$\lim_{\theta \to \infty} \psi_\theta(t) = \varsigma(t)$, $\forall t \in \mathbb{R}$. For the sake of simplicity, we will use ψ instead of ψ_θ. We suppose that ψ is a DC function, i.e.,

$$\psi(t) := \vartheta(t) - \omega(t)$$

where ϑ and ω are convex functions. Hence, the ℓ_0 norm of a vector s can be approximated by

$$\|s\|_0 = \sum_{i=1}^{n} \varsigma(s_i) \approx \sum_{i=1}^{n} \psi(s_i) =: \alpha(s) - \beta(s) \tag{23}$$

with $\alpha(s) := \sum_{i=1}^{n} \vartheta(s_i)$ and $\beta(s) := \sum_{i=1}^{n} \omega(s_i)$. Since ϑ and ω are convex, α and β are also convex. Therefore, $\|s\|_0$ can be approximated by a DC function.

Using the formula (23), we approximate the original Problem (21) with

$$\begin{aligned}
\min_{x,y,z} \quad & \zeta(x,y,z) := c^T \begin{bmatrix} x \\ y \\ z \end{bmatrix} + \lambda\left(\alpha(x) - \beta(x)\right) - \delta\left(\alpha(y) - \beta(y)\right) \\
\text{s.t.} \quad & A \begin{bmatrix} x \\ y \\ z \end{bmatrix} \leq b, \\
& l \leq \begin{bmatrix} x \\ y \\ z \end{bmatrix} \leq u, \\
& x \in \mathbb{R}^p,\ y \in \mathbb{R}^q,\ z \in \mathbb{R}^r
\end{aligned} \tag{24}$$

The objective function $\zeta(x,y,z)$ can be rewritten as

$$\zeta(x,y,z) := \phi(x,y,z) - \varphi(x,y,z)$$

where $\phi(x,y,z) := c^T \begin{bmatrix} x \\ y \\ z \end{bmatrix} + \lambda\alpha(x) + \delta\beta(y)$ and $\varphi(x,y,z) := \lambda\beta(x) + \delta\alpha(y)$ are clearly convex functions. Hence $\zeta(x,y,z)$ is a DC function. Based on the generic algorithm given in Section 1.2.1, the algorithm we apply to Problem (24) is:

Difference of Convex Cardinality Optimisation (DCCO) Algorithm:

0. Consider Problem (24). Set a numerical stopping criterion ϵ.
1. Let (x^0, y^0, z^0) be any initial point andϵ be a sufficiently small tolerance. Set $k = 0$.
2. Compute $(\tilde{x}^k, \tilde{y}^k, \tilde{z}^k)$ which belongs to the subdifferential of φ at (x^k, y^k, z^k):$(\tilde{x}^k, \tilde{y}^k, \tilde{z}^k) \in \partial\varphi\left(x^k, y^k, z^k\right)$.
3. Compute $(x^{k+1}, y^{k+1}, z^{k+1})$ by solving the convex optimisation problem, denoted $\mathcal{P}_k$:

$$\begin{aligned}
\min_{x,y,z} \quad & c^T \begin{bmatrix} x \\ y \\ z \end{bmatrix} + \lambda\alpha(x) + \delta\beta(y) - x^T\tilde{x}^k - y^T\tilde{y}^k - z^T\tilde{z}^k \\
\text{s.t.} \quad & A \begin{bmatrix} x \\ y \\ z \end{bmatrix} \leq b \\
& l \leq \begin{bmatrix} x \\ y \\ z \end{bmatrix} \leq u \\
& x \in \mathbb{R}^p,\ y \in \mathbb{R}^q,\ z \in \mathbb{R}^r
\end{aligned} \tag{25}$$

4. If $\|(x^{k+1}, y^{k+1}, z^{k+1}) - (x^k, y^k, z^k)\| < \epsilon$ or$\|\zeta(x^{k+1}, y^{k+1}, z^{k+1}) - \zeta(x^k, y^k, z^k)\| < \epsilon$ then STOP and RETURN $(x^{k+1}, y^{k+1}, z^{k+1})$, otherwise set $(x^k, y^k, z^k) = (x^{k+1}, y^{k+1}, z^{k+1})$, set $k = k + 1$, and go to Step 2.

1.2.3 Approximate step functions

Many approximate step functions are possible, but we focus on the following continuous approximate step functions: the piece-wise exponential function (ψ_{exp}, [13]), the smoothly clipped absolute deviation penalty function (ψ_{SCAD}, [14]), the logarithm function (ψ_{log}, [15]), the ℓ_{p+} norm with $0 < p < 1$ ($\psi_{\ell_p^+}$, [16]), the ℓ_{p-} norm with $p < 0$ ($\psi_{\ell_p^-}$, [17]), and the capped $-\ \ell_1$ function (ψ_{cap}, [18]), all illustrated in Figure 4. Each of the aforementioned approximations may be represented as a DC function [9]. The definition of each approximate step

function and their DC decompositions are given in Table 1. For each approximate step function, Supplementary Table 2 gives the sub-gradients of their DC components, ϑ and ω.

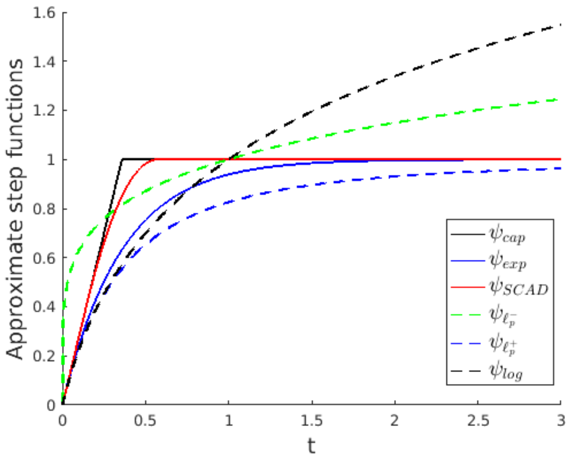

Figure 4: **Approximate step functions**. ψ_{log} and $\psi_{\ell_p^-}$ tend to $+\infty$ when t tends to $+\infty$, while ψ_{exp},ψ_{SCAD},$\psi_{\ell_p^+}$, and ψ_{cap} are step function minorants and the greater t is, the closer to the value of a step function they become. Based on Fig 1 in [9].

Table 1: Approximate step functions and their decomposition into a difference of convex functions $\psi(t) = \vartheta(t) - \omega(t)$. Each approximate step function depends on a step parameter θ that governs the fidelity of the approximation.

	**Approximate step function**	**Convex functions**	
	$\psi(t)$	$\vartheta(t)$	$\omega(t)$
Piecewise exponential	$\psi_{exp}(t) = 1 - e^{-\theta\|t\|}$	$\theta\|t\|$	$\theta\|t\| - 1 + e^{-\theta\|t\|}$
Smoothly clipped absolute deviation	$\psi_{\mathrm{scad}}(\mathrm{t}) = \begin{cases} \frac{2\theta}{a+1}\|t\| & if\ 0 \le \|t\| \le \frac{1}{\theta} \\ \frac{-\theta^2 t^2 + 2a\theta\|t\| - 1}{a^2-1} & if\ \frac{1}{\theta} < \|t\| < \frac{a}{\theta} \\ 1 & if\ \|t\| \ge \frac{a}{\theta} \end{cases}$	$\dfrac{2\theta}{\mathrm{a}+1}\|\mathrm{t}\|$	$\begin{cases} 0 & if\ 0 \le \|t\| \le \frac{1}{\theta} \\ \frac{-(\theta\|t\|+1)^2}{a^2-1} & if\ \frac{1}{\theta} < \|t\| < \frac{a}{\theta} \\ \dfrac{2\theta}{a+1}\|t\| - 1 & if\ \|t\| \ge \frac{a}{\theta} \end{cases}$
Logarithm	$\psi_{log}(t) = \frac{\log(1+\theta\|t\|)}{\log(1+\theta)}$	$\frac{\theta}{\log(1+\theta)}\|\mathrm{t}\|$	$\frac{\theta\|t\| - \log(1+\theta\|t\|)}{\log(1+\theta)}$
ℓ_{p+} norm with$0 < p < 1$	$\psi_{\ell_\mathrm{p}^+}(\mathrm{t}) = (\|\mathrm{t}\| + \epsilon)^{1/\theta}$	$\frac{\epsilon^{1/\theta - 1}}{\theta}\|\mathrm{t}\|$	$\frac{\epsilon^{1/\theta-1}}{\theta}\|t\| - (\|t\| + \epsilon)^{1/\theta}$
ℓ_{p-} norm with$p < 0$	$\psi_{\ell_\mathrm{p}^-}(\mathrm{t}) = 1 - (1 + \theta\|\mathrm{t}\|)^\mathrm{p}, \mathrm{p} < 0$	$-\mathrm{p}\theta\|\mathrm{t}\|$	$(1 + \theta\|t\|)^p - p\theta\|t\| - 1$
Capped-ℓ_1	$\psi_{\mathrm{cap}}(\mathrm{t}) = \min\{1, \theta\|\mathrm{t}\|\}$	$\theta\|\mathrm{t}\|$	$\max\{1, \theta\|t\|\} - 1$

1.2.4 Convergence properties

Based on the convergence properties of difference of convex function algorithms, certain aspects of the convergence of the DCCO algorithm are assured. Specifically, it generates a sequence$(\tilde{x}^k, \tilde{y}^k, \tilde{z}^k)$ such that the sequence $\zeta(x^k, y^k, z^k)$ is monotonically decreasing and the sequence (x^k, y^k, z^k) globally converges to (x^*, y^*, z^*), a critical point of the objective function ζ ([12], Theorem 3). Furthermore, when the approximate step function is the capped-ℓ_1 norm, the Problem (24) is a polyhedral DC, i.e, the DC components are a polyhedral convex functions. Hence, the DCCO algorithm has finite convergence. Moreover, if the function $\varphi(x, y, z)$ is differentiable at (x^*, y^*, z^*), then (x^*, y^*, z^*) is a local solution of (24) (Theorem 5, [12]). For a sufficiently large parameter θ, the Capped-ℓ_1 approximate problem and the original cardinality optimisation problem are have the same set of optimal solutions [9]. Moreover, the DCCO with a capped-ℓ_1 approximation, will converge to a local optimum while the other approximations will only converge to a critical point [9].

1.2.5 Inner convex optimisation iteration assuming a Capped-l_1 approximation

Consider the cardinality optimisation problem 21, and assume the zero norm is represented by a capped-ℓ_1 step function, that is $\psi(\mathrm{t}) = \psi_{\mathrm{cap}}(\mathrm{t}) := \min\{1, \theta\|\mathrm{t}\|\}$,then the objective function in 21 may be approximated by

Table 2: The sub-gradients of the convex functions ϑ and ω, where ϑ and ω are the components of the difference of convex function ψ.

Approximation	$\partial\vartheta(\mathrm{t})$	$\partial\omega(t)$
Piecewise exponential	$\mathrm{sign(t)}\theta$	$\mathrm{sign}(t)\theta\left(1-e^{-\theta\lvert t\rvert}\right)$
SCAD	$\mathrm{sign(t)}\dfrac{2\theta}{\mathrm{a}+1}$	$\begin{cases} 0 & if\ \lvert t\rvert \leq \frac{1}{\theta} \\ \mathrm{sign}(t)\frac{2\theta(\theta\lvert t\rvert+1)}{a^2-1} & if\ \frac{1}{\theta} < \lvert t\rvert < \frac{a}{\theta} \\ \mathrm{sign}(t)\frac{2\theta}{a+1} & \text{if}\ \lvert\mathrm{t}\rvert \geq \frac{a}{\theta} \end{cases}$
Logarithm	$\mathrm{sign(t)}\frac{\theta}{\log(1+\theta)}$	$\mathrm{sign(t)}\frac{\lambda\theta^2\lvert\mathrm{s}\rvert}{\log(1+\theta)(1+\theta\lvert\mathrm{s}\rvert)}$
ℓ_{p+} norm with$0<p<1$	$\mathrm{sign(t)}\frac{\epsilon^{1/\theta-1}}{\theta}$	$\mathrm{sign(t)}\frac{1}{\theta}\left[\epsilon^{1/\theta-1}-(\lvert\mathrm{t}\rvert+\epsilon)^{1/\theta-1}\right]$
ℓ_{p-} norm with$p<0$	$-\mathrm{p}\theta\mathrm{sign(t)}$	$-\mathrm{sign}(t)p\theta\left[1-(1+\theta\lvert t\rvert)^{p-1}\right]$
Capped-ℓ_1	$\mathrm{sign(t)}\theta$	$\begin{cases} 0 & if\ \lvert t\rvert \leq \frac{1}{\theta} \\ \mathrm{sign}(t)\theta & \text{otherwise} \end{cases}$

$$c^T\begin{bmatrix} x \\ y \\ z \end{bmatrix} + \lambda\|x\|_0 - \delta\|y\|_0 \approx c^T\begin{bmatrix} x \\ y \\ z \end{bmatrix} + \lambda\sum_{i=1}^{p}\min\{1,\theta\lvert x_i\rvert\} - \delta\sum_{i=1}^{q}\min\{1,\theta\lvert y_i\rvert\}.$$

The capped-ℓ_1 step function may be expressed as the difference between two convex functions $\psi(t) := \vartheta(t) - \omega(t)$,where $\vartheta(t) = \theta\lvert\mathrm{t}\rvert$ and $\omega(t) = \max\{1, \theta\lvert t\rvert\} - 1$, then $\psi_{cap}(t) = \theta\lvert t\rvert - \max\{1, \theta\lvert t\rvert\} - 1$. Therefore, the objective in 24 becomes

$$\begin{aligned}
\zeta(x,y,z) &:= c^T\begin{bmatrix} x \\ y \\ z \end{bmatrix} + \lambda\left(\alpha(x)-\beta(x)\right) - \delta\left(\alpha(y)-\beta(y)\right) \\
&= c^T\begin{bmatrix} x \\ y \\ z \end{bmatrix} + \lambda\sum_{i=1}^{p}\left(\theta\lvert x_i\rvert - (\max\{1,\theta\lvert x_i\rvert\}-1)\right) - \delta\sum_{i=1}^{q}\left(\theta\lvert y_i\rvert - (\max\{1,\theta\lvert y_i\rvert\}-1)\right) \\
&= c^T\begin{bmatrix} x \\ y \\ z \end{bmatrix} + \left(\lambda\sum_{i=1}^{p}\theta\lvert x_i\rvert + \delta\sum_{i=1}^{q}(\max\{1,\theta\lvert y_i\rvert\}-1)\right) - \left(\lambda\sum_{i=1}^{p}(\max\{1,\theta\lvert x_i\rvert\}-1) + \delta\sum_{i=1}^{q}\theta\lvert y_i\rvert\right)
\end{aligned}$$

which is a difference of convex functions of the form $\zeta(x,y,z) := \phi(x,y,z) - \varphi(x,y,z)$ since

$$\begin{aligned}
\phi(x,y,z) &:= c^T\begin{bmatrix} x \\ y \\ z \end{bmatrix} + \left(\lambda\sum_{i=1}^{p}\theta\lvert x_i\rvert + \delta\sum_{i=1}^{q}(\max\{1,\theta\lvert y_i\rvert\}-1)\right) \\
\varphi(x,y,z) &:= \left(\lambda\sum_{i=1}^{p}(\max\{1,\theta\lvert x_i\rvert\}-1) + \delta\sum_{i=1}^{q}\theta\lvert y_i\rvert\right)
\end{aligned}$$

are both convex functions.

The subdifferentials of φ at (x^k, y^k, z^k) are denoted $(\tilde{x}^k, \tilde{y}^k, \tilde{z}^k) \in \partial\varphi\left(x^k, y^k, z^k\right)$ and for the capped-ℓ_1 step function they are

$$\begin{aligned}
\tilde{x_i}^k = \frac{\partial\varphi\left(x^k,y^k,z^k\right)}{\partial x_i} &= \begin{cases} 0 & if\ \lvert x_i\rvert \leq \frac{1}{\theta} \\ \mathrm{sign}(x_i^k)\theta & \text{otherwise} \end{cases} \\
\tilde{y_i}^k = \frac{\partial\varphi\left(x^k,y^k,z^k\right)}{\partial y_i} &= \mathrm{sign}(y_i^k)\theta \\
\tilde{z_i}^k = \frac{\partial\varphi\left(x^k,y^k,z^k\right)}{\partial z_i} &= 0.
\end{aligned}$$

Therefore, in Step 4 of the Difference of Convex Cardinality Optimisation (DCCO) Algorithm, the inner convex optimisation problem is used to obtain $(x^{k+1}, y^{k+1}, z^{k+1})$ by equating it to the solution of the convex

optimisation 25, with objective

$$
\begin{aligned}
\phi(x,y,z)-\begin{bmatrix} x\\ y\\ z\end{bmatrix}^T \begin{bmatrix} \tilde{x}^k\\ \tilde{y}^k\\ \tilde{z}^k\end{bmatrix} &= c^T\begin{bmatrix} x\\ y\\ z\end{bmatrix} + \left(\lambda\sum_{i=1}^{p}\theta|x_i| + \delta\sum_{i=1}^{q}(\max\{1,\theta|y_i|\}-1)\right)\\
&\dots\ -\lambda\sum_{i=1}^{p}\left(x_i\times\tilde{x}_i^k\right) - \delta\sum_{i=1}^{q}\left(y_i\times\tilde{y}_i^k\right),\\
&= c^T\begin{bmatrix} x\\ y\\ z\end{bmatrix} + \left(\lambda\sum_{i=1}^{p}\theta|x_i| + \delta\sum_{i=1}^{q}(\max\{1,\theta|y_i|\}-1)\right)\\
&\dots\ -\lambda\sum_{i=1}^{p}\left(x_i\times\tilde{x}_i^k\right) - \delta\sum_{i=1}^{q}\left(y_i\times\text{sign}(y_i^k)\theta\right).
\end{aligned}
$$

This discontinuous objective may be reformulated into a continuous objective by letting $w_i := max\{x_i, -x_i\} \geq 0$ and $t_i := max\{1, \theta y_i\} \geq 1$ so Problem 25 becomes

$$
\begin{aligned}
\min_{x,y,z}\quad & c^T\begin{bmatrix} x\\ y\\ z\end{bmatrix} + \left(\lambda\sum_{i=1}^{p}\theta|w_i| + \delta\sum_{i=1}^{q}(t_i-1)\right)\\
& -\lambda\sum_{i=1}^{p}\left(x_i\times\tilde{x}_i^k\right) - \delta\sum_{i=1}^{q}\left(y_i\times\text{sign}(y_i^k)\theta\right)\\
\text{s.t.}\quad & A\begin{bmatrix} x\\ y\\ z\end{bmatrix} \leq b,\\
& l \leq \begin{bmatrix} x\\ y\\ z\end{bmatrix} \leq u,\\
& x - w \leq 0,\\
& -x - w \leq 0,\\
& \theta y - t \leq 0,\\
& -\theta y - t \leq 0,\\
& x\in\mathbb{R}^p,\ y\in\mathbb{R}^q,\ z\in\mathbb{R}^r,
\end{aligned}
\tag{26}
$$

which is a linear optimisation problem subject to linear constraints.

1.3 Computational implementation

1.3.1 Parametrisation

Theoretically, the greater the value of step parameter θ in Table (1), the better the approximation of a step function. However, practically, a greater value of θ, will lead to more local minima of the approximate cardinality optimisation problem. Hence, we use the following parameter updating procedure. Starting with a small value θ_0, the parameter θ was gradually increased until a given threshold θ_{max}. In our experiments, θ_0 was set to 0.5 and θ_{max} was equal to 100. The tolerance for the stopping criterion of the DCCO algorithm was set with $\epsilon = 10^{-6}$. An upper bound of 10^9 was set on each w_i in Problem (1 ,main text) to avoid excessively large numerical values. The threshold to distinguish non-zero and zero v_j in Problem (19) was set with $\varepsilon = 10^{-8}$ as it is 100 times less than the (default) feasibility tolerance (10^{-6}) of the linear optimisation solver. Note that, in practice setting ε to be equal the feasibility tolerance of the linear optimisation solver is too stringent, since some nonzero values that smaller in magnitude (e.g. between10^{-8} and10^{-6}) are necessary to satisfy the optimality conditions with a residual less than ε for a typical stoichiometric matrix, with coefficients of order one.

1.3.2 Hardware and software

The interface to each optimisation problem, as well as the the DCCO algorithm were implemented in MATLAB (Mathworks Inc) and integrated with the COBRA toolbox [19], a comprehensive, cross-platform software suite for constraint-based modelling of biochemical networks, available at https://github.com/opencobra/cobratoolbox. The optimizeCardinality.m function is the general purpose interface to solve Problem 21 using our implementation of the Difference of Convex Cardinality Optimisation (DCCO) Algorithm described in Section 1.2.2. Stoichiometric consistency testing is implemented by the function findStoichConsistentSubset.m, leak and siphon testing is implemented by the function findMassLeaksAndSiphons.m, flux consistency testing is implemented by the function findFluxConsistentSubset.m, thermodynamic flux consistency testing is implemented by

the function findThermoConsistentFluxSubset.m, sparse flux balance analysis is implemented by the function sparseFBA.m and relaxed flux balance analysis is implemented by the function relaxedFBA.m. The function checkModelProperties.m provides a single interface that analyses leaks/siphons as well as stoichiometric, flux, thermodynamic flux, and a previously developed implementation that analyses a form of kinetic consistency [3]. Scripts to reproduce the figures and tables are available at https://github.com/opencobra/COBRA.papers/2022_cardOpt. Furthermore, a set of tutorials illustrating each cardinality optimisation application are also available within the COBRA toolbox tutorial collection (https://github.com/opencobra/COBRA.tutorials). The COBRA toolbox provides interfaces to a wide range of commercial and open-source optimisation solvers, that can be selected to solve the optimisation subproblems that arise at each iteration of the DCCO algorithm. Numerical timing experiments were performed with MATLAB version R2021b on a desktop Intel Core i5 @3.30GHz with 16GB RAM with a 64-bit Ubuntu 20.04 operating system. Gurobi Optimizer 6.0 (Gurobi Optimization, Inc.) was used to numerically solve all linear and mixed-integer linear sub-problems, with the exception of Supplementary Table 4, where IBM CPLEX 12.0 was used.

2 Supplementary results

2.1 Stoichiometric consistency

Supplementary Table 3 compares the performance of three approaches for computing the maximal cardinality conservation vector (Problem (6)) and two approaches for computing the minimal cardinality conservation relaxation vector (Problem 1, main text) for a selection of 7 metabolic networks. In Supplementary Table 3, the *MILP formulation* approximately solves Problem (6) using the Mixed Integer Linear Program given by Problem (7). The *Linear approx.* approximately solves 6 using the linear optimisation problem given by Problem (8). *Capped-ℓ_1DCCO* approximately solves 6 using the difference of convex function cardinality optimisation algorithm described in Supplementary Section 1.2. The maximal number of conserved metabolites and reactions identified is the same for both the MILP formulation and the Capped-ℓ_1DCCO approach, and both are substantially larger than for the linear approximation, unless all of the internal reactions of the network are stoichiometrically consistent, in which case all three report the same result. This demonstrates that, despite the fact that Capped-ℓ_1DCCO approach solves an approximation to Problem (6), it returns the same number of metabolites and reactions as the solution to the mixed-integer linear program.

In Supplementary Table 3, *Sequential capped-ℓ_1DCCO (omit mass imbalanced)* implements a sequence of iterates, each of Problem 1 (main text) solved using the difference of convex function cardinality optimisation algorithm described in Supplementary Section 1.2, and after each iterate it omits elementally imbalanced reactions with $x^\star \neq 0$, and the metabolites exclusively involved in those reactions. *Sequential capped-ℓ_1DCCO (omit lumped reactions)* implements a sequence of iterates, each of Problem 1 (main text) solved using the difference of convex function cardinality optimisation algorithm described in Supplementary Section 1.2, and after each iterate it omits lumped reactions (with the highest number of stoichiometric coefficients) and with $x^\star \neq 0$, and the metabolites exclusively involved in those reactions. Both of these approaches report that the stoichiometrically consistent subset is substantially larger for every network than the largest subnetwork reported by either the MILP formulation, the Capped-ℓ_1DCCO approach or the linear approximation. This demonstrates the importance of omitting reactions of unknown but suspected stoichiometric consistency, in revealing a larger stoichiometrically consistent subset than with the solution to a single optimisation problem alone.

Supplementary Table 3 also provides a comparison of total runtimes for various approaches. This demonstrates that sequential capped-ℓ_1DCCO is substantially slower than other approaches, but it does always identify the largest stoichiometrically consistent subset for all networks. Supplementary Table 3 is not a definitive comparison of MILP versus capped-ℓ_1DCCO solution times, since a substantial subset of the reported time is required for overhead, e.g., setting the solver in the COBRA toolbox, testing for stoichiometric consistency before deciding whether to find the largest stoichiometrically consistent subset, testing the optimality of computed solutions, etc. Therefore, supplementary Table 4 provides a comparison of the time taken, by MILP and capped-ℓ_1DCCO, to compute maximal cardinality conservation vectors for a range of networks, where overhead has been reduced to a minimum. Each capped-ℓ_1DCCO problem is solved by a sequence of linear optimisation problems. The results presented include a substantial speed up achieved by warm starting the second and subsequent linear optimisations with the optimal solution of the previous optimisation. While MILP is consistently faster for smaller networks, Capped-ℓ_1DCCO is consistently faster for the largest networks tested, that is the female and male whole body metabolic models [26].

2.2 Flux consistency

In practice, for this instance of cardinality maximisation alone, rather than a difference of convex function approach, we observed that it was more efficient to employ the FASTCORE algorithm [5], implemented within

Table 3: **Comparison of five approaches to identify the largest stoichiometrically consistent subset.** For each test network, the identity, citation and number of heuristically internal metabolites (m) and reactions (n) is provided. For each algorithm, the number of stoichiometrically consistent metabolites m^* and reactions n^* is reported, as well as the runtime in seconds. The largest stoichiometric consistency results are emphasised in bold font.

Networks \ Algorithms				**MILP formulation**			**Linear approx.**			**Capped-ℓ_1DCCO**			**Sequential capped-ℓ_1DCCO** (omit mass imbalanced)			**Sequential capped-ℓ_1DCCO** (omit lumped reactions)		
ID	Ref.	m	n	m^*	n^*	Sec.	m^*	n^*	Sec.	m^*	n^*	Sec.	m^*	n^*	Sec.	m^*	n^*	Sec.
iAC560	[20]	1101	1048	563	555	37.1	148	209	1.3	563	555	1.0	**1101**	**1021**	10.9	**1101**	**1021**	5.8
iFF708	[21]	794	1167	417	712	4.7	322	684	0.40	417	712	0.5	793	**1085**	8.4	**794**	1055	1.5
iLC915	[22]	1300	1365	454	367	0.3	80	72	0.4	454	367	0.2	**1300**	**1303**	11.1	1300	1299	5.2
iRS1563	[23]	2086	1766	1254	966	0.3	305	308	0.4	1254	966	0.3	2086	1710	4.4	**2086**	**1712**	5.1
Recon3.01	[24]	8396	11646	1661	2665	0.3	1529	2328	0.5	1661	2665	0.6	**8380**	**11345**	1296.6	8384	11079	6029.2
Recon3.01model	[24]	5835	8791	**5835**	**8791**	0.3	**5835**	**8791**	0.3	**5835**	**8791**	0.3	**5835**	**8791**	0.6	**5835**	**8791**	0.3
Human1.0	[25]	8422	11795	1831	2733	24.3	363	1108	4.4	1831	2733	17.6	8408	10944	700.1	**8418**	**10954**	2283.2

Table 4: **Temporal performance comparison for MILP versus capped-ℓ_1DCCO.** The maximal cardinality conservation vector was computed for a range of networks using MILP and capped-ℓ_1DCCO, and IBM CPLEX 12.0 as the MILP and LP solver. For each test network, the identity, citation and number of heuristically internal metabolites (m) and reactions (n) is provided. For each algorithm, mean and standard deviation of the solution time is given in seconds (s). The average fastest approach for each network are emphasised in bold font.

Networks \ Algorithms				**MILP**		**Capped-ℓ_1DCCO**	
ID	Ref.	m	n	Mean (s)	Standard deviation (s)	Mean (s)	Standard deviation (s)
iAC560	[20]	1101	1048	**0.14899**	0.041743	0.20828	0.015381
iFF708	[21]	794	1167	**0.073813**	0.012158	0.13727	0.0043773
iLC915	[22]	1300	1365	**0.10352**	0.011621	0.21963	0.0099388
iRS1563	[23]	2086	1766	**0.12283**	0.015257	0.30398	0.017278
Recon3.01	[24]	8396	11646	**0.12603**	0.018644	0.21901	0.022738
Recon3.01model	[24]	5835	8791	**0.44115**	0.13621	0.48502	0.064534
Human1.0	[25]	8422	11795	**1.6502**	0.17039	8.0795	7.3977
Harvetta	[26]	58808	78539	18.028	1.3571	**12.073**	1.1428
Harvey	[26]	56411	76243	15.3433	0.4142	**10.6288**	0.1690

the COBRA toolbox [27].

2.3 Sparse flux balance analysis

Table 5 provides a comparison of numerical results for flux balance analysis (FBA), one norm minimisation ($\ell_1$min) and sparse flux balance analysis with cardinality optimisation approximated by minimisation of the difference of convex functions (DCCO). Table 6 illustrates a stoichiometrically balanced cycle involving ATP hydrolysis in Recon3D.

Table 5: Comparison of numerical results for flux balance analysis (FBA), one norm minimisation ($\ell_1$min) and cardinality optimisation approximated by minimisation of the difference of convex functions (DCCO). Herein, n^* denotes the number of predicted active reactions and k denotes the number of predicted active reactions that could be removed from the solution, yet satisfy the optimal objective of FBA. The best step function approximation reported by the DCCO algorithm is usually the Capped-ℓ_1 approximation.

Data				FBA(9)		$\ell_1$min [28]		DCCO with all approx. (19)				
Model name	Ref	m	n	n^*	time(s)	n^*	time(s)	Best approx.	n^*	time(s)	#LPs	k
iIB711	[29]	496	971	343	**0.02**	243	0.02	SCAD	**233**	0.05	2	6
iJN746	[30]	909	1,056	395	**0.04**	357	0.06	Capped-ℓ_1	**346**	0.10	3	0
iND750	[31]	1,061	1,266	324	**0.02**	**279**	0.04	ℓ_{p-}	**279**	0.08	2	0
iCac802	[32]	1,253	1,462	327	**0.03**	**320**	0.08	ℓ_{p+}	321	0.05	2	0
iMM904	[33]	1,226	1,577	341	**0.05**	302	0.12	Capped-ℓ_1	**298**	0.13	2	0
Harvetta	[26]	58,851	83,521	22,252	**30.04**	9,601	73.67	Capped-ℓ_1	**5,131**	532.5	20	41

Table 6: A stoichiometrically balanced cycle involving ATP hydrolysis in Recon3D. No stoichiometrically balanced cycle contributing to isolated ATP hydrolysis (bottom row, same as reaction (5) in the main text) is present in Recon3Dmodel. All reaction and metabolite abbreviations are primary keys in the Virtual Metabolic Human Database, accessible at vmh.life. †This reaction is irreversible in the forward direction in Recon3Dmodel, thus eliminating isolated ATP hydrolysis in this cycle.

**Reaction**	**Recon3D Reaction equation with metabolite abbreviations**	**Net flux**
PYNP2r	pi[c] + uri[c] <=> ura[c] + r1p[c]	1000
r1156	uri[c] + dutp[c] <=> h[c] + dudp[c] + ump[c]	-1000
RE3239C	h2o[c] + CE5155[c] <=> h[c] + coa[c] + doco13ac[c]	-1000
NDPK6	atp[c] + dudp[c] <=> adp[c] + dutp[c]	-1000
PPM	r1p[c] <=> r5p[c]	1000
PRPPS	atp[c] + r5p[c] <=> h[c] + amp[c] + prpp[c]	1000
†HMR_0319	atp[c] + coa[c] + doco13ac[c] <=> amp[c] + ppi[c] + CE5155[c]	-1000
HMR_4343	ura[c] + prpp[c] <=> ppi[c] + ump[c]	1000
DM_atp_c_	h2o[c] + atp[c] <=> h[c] + adp[c] + pi[c]	1000

2.4 Relaxed Flux Balance Analysis

2.4.1 A toy example

Consider the toy metabolic network in Figure (27), which contains 8 metabolites (labelled A,. . .,H) and 11 unidirectional reactions, with corresponding fluxes $v_1, \ldots, v_{11}$, represented by an 8×11 stoichiometric matrix, S. The set of upper bound and lower bound of reactions is denoted
$\mathfrak{B} := \left\{v \in \mathbb{R}^{11} | 0 \leq v_i \leq 10,\ i = 1 \ldots 6, 8 \ldots 10;\ 1 \leq v_7 \leq 10\right\}$. It is clear that metabolite E is always consumed but never produced and therefore there exists no $v \in \mathfrak{B}$ that satisfies $Sv = 0$. Relaxed flux balance analysis can be used to find a minimal number of internal reaction bounds to relax if one excludes exchange reactions, by fixing the relaxation on lower bound and upper bound of fluxes $v_1$and v_7 to 0, i.e., $p_1 = p_7 = q_1 = q_7 = 0$. Three solutions exist to relax the bounds in order to make the network feasible. The first solution is to allow reaction flux v_5 to be a reversible reaction by relaxing its lower bound. In the second solution (respectively, third solution), one needs to relax the bounds on two reaction fluxes v_4 andv_6 (respectivelyv_9,v_{10}, andv_{11}). Consequently, the first solution is chosen since the number of relaxations needed is smallest.

We apply our algorithm to find the minimum number of relaxations needed to make the toy network feasible. Using DCCO with a capped-ℓ_1 approximation to solve the following problem

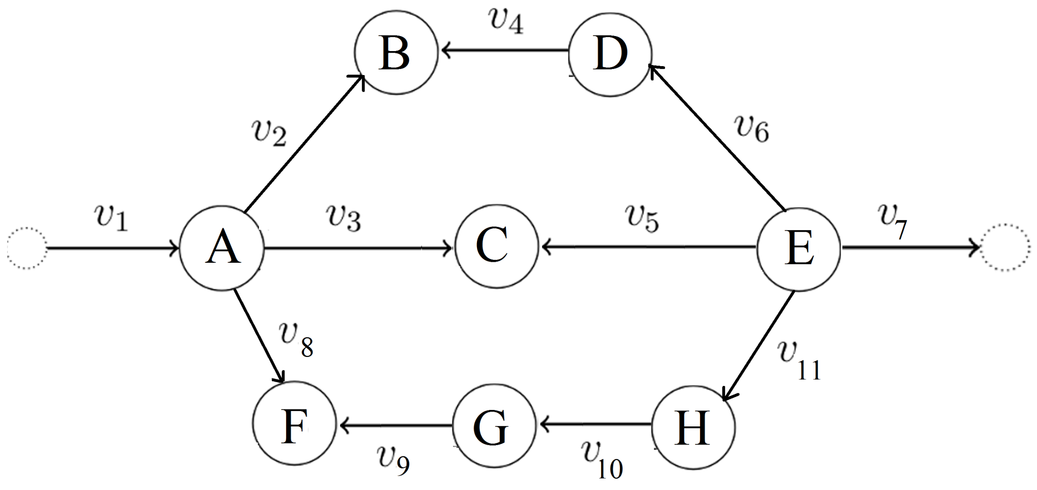

Figure 5: **A toy biochemical network.** We assume that all reactions are irreversible. The fluxes are bounded such that $0 \leq v_i \leq 10,\ i = 1 \dots 10$, except for the reaction r_7 with bounds $1 \leq v_7 \leq 10$.

$$
\begin{array}{ll}
\min\limits_{v,r,p,q} & \|r\|_0 + \|p\|_0 + \|q\|_0 \\
\text{s.t.} & Sv + r = 0 \\
& l - p \leq v \leq u + q \\
& p_1 = p_7 = q_1 = q_7 = 0 \\
& p, q \in \mathbb{R}_+^{11} \\
& r \in \mathbb{R}_+^{8}
\end{array}
\tag{27}
$$

the solution (v^*, p^*, q^*, r^*) given is $q^* = 0$,$r^* = 0$ and $p_5^* = 10$; $p_i^* = 0,\ i = 1 \dots 4, 6 \dots 11$. This means that only the lower bound of reaction r_5 needs to be relaxed in order to make the biochemical network feasible. This result perfectly corresponds to the anticipated solution. In a second setting, we disallow the relaxation of the bound on reaction r_5. As expected, the best possible solution, to only relax the bounds of the reactions r_4 and r_6, is returned.

Table 7: Starting with Recon3Dmodel, then setting all external reaction bounds to zero, yet requiring a biomass reaction (VMH biomass_reaction https://vmh.life/#reaction/biomass_reaction) rate between a lower bound of 1 and an upper bound of 10 created an infeasible model. With RelaxedFBA, to enable biomass production, a minimal cardinality solution required the relaxation of 24 lower bounds and 2 upper bounds.

**External reaction abbreviation**	**Relaxation of lower bound**
DM_datp_n_	0.112826
DM_dctp_n_	0.101924
DM_dttp_n_	0.0615923
EX_crvnc[e]	0.0805026
EX_pchol_hs[e]	0.017486
EX_spc_hs[e]	0.03643
EX_strch1[e]	0.110829
EX_xolest226_hs[e]	0.020401
EX_cysleuthr[e]	0.046571
EX_hislysval[e]	0.317637
EX_ileprolys[e]	0.137501
EX_leuleutrp[e]	0.013306
EX_lysargleu[e]	0.297908
EX_lyslyslys[e]	0.286568
EX_lystyrile[e]	0.101269
EX_metmetile[e]	0.0473079
EX_pheleuhis[e]	0.139482
EX_phethrlys[e]	0.0850133
EX_thrthrarg[e]	0.0613517
EX_thrtyrmet[e]	0.0584023
EX_valleuphe[e]	0.0349704
DM_mi1345p[c]	0.023315
DM_pchol_hs[c]	0.265211
EX_o2[e]	1000

**External reaction abbreviation**	**Relaxation of upper bound**
EX_2oxoadp[e]	1000
EX_2m3hvac[e]	0.0650387

References

[1] A. Gevorgyan, M. G. Poolman and D. A. Fell. “Detection of Stoichiometric Inconsistencies in Biomolecular Models.” In: *Bioinformatics* 24.19 (2008), pp. 2245–51.

[2] E. V. Nikolaev, A. P. Burgard and C. D. Maranas. “Elucidation and Structural Analysis of Conserved Pools for Genome-Scale Metabolic Reconstructions.” In: *Biophysical Journal* 88.1 (2005), pp. 37–49.

[3] R. M. T. Fleming et al. “Conditions for Duality between Fluxes and Concentrations in Biochemical Networks”. In: *Journal of Theoretical Biology* 409 (Nov. 2016), pp. 1–10.

[4] I. Thiele and B. Ø. Palsson. “A Protocol for Generating a High-Quality Genome-Scale Metabolic Reconstruction.” In: *Nature Protocols* 5.1 (2010), pp. 93–121.

[5] N. Vlassis, M. P. Pacheco and T. Sauter. “Fast Reconstruction of Compact Context-Specific Metabolic Network Models”. In: *PLoS Comput Biol* 10.1 (Jan. 2014), e1003424.

[6] A. A. Desouki et al. “CycleFreeFlux: Efficient Removal of Thermodynamically Infeasible Loops from Flux Distributions”. In: *Bioinformatics* 31.13 (July 2015), pp. 2159–2165.

[7] R. M. T. Fleming et al. “A Variational Principle for Computing Nonequilibrium Fluxes and Potentials in Genome-Scale Biochemical Networks.” In: *Journal of Theoretical Biology* 292 (2011), pp. 71–77.

[8] M. Tefagh and S. P. Boyd. “SWIFTCORE: A Tool for the Context-Specific Reconstruction of Genome-Scale Metabolic Networks”. In: *BMC Bioinformatics* 21.1 (Apr. 2020), p. 140.

[9] H. A. Le Thi et al. “DC Approximation Approaches for Sparse Optimization”. In: *European Journal of Operational Research* 244.1 (July 2015), pp. 26–46.

[10] E. Meléndez-Hevia and A. Isidoro. “The Game of the Pentose Phosphate Cycle”. In: *Journal of Theoretical Biology* 117.2 (1985), pp. 251–263.

[11] H. Le Thi and T. Pham Dinh. “The DC (Difference of Convex Functions) Programming and DCA Revisited with DC Models of Real World Nonconvex Optimization Problems”. In: *Annals of Operations Research* 133.1-4 (Jan. 2005), pp. 23–46.

[12] T. Pham Dinh and H. Le Thi. “Convex Analysis Approach to DC Programming: Theory, Algorithms and Applications”. In: *Acta Mathematica Vietnamica* 22.1 (1997), pp. 289–355.

[13] P. S. Bradley and O. L. Mangasarian. “Feature Selection via Concave Minimization and Support Vector Machines”. In: *Machine Learning Proceedings of the Fifteenth International Conference.* ICML ’98. Morgan Kaufmann, 1998, pp. 82–90.

[14] J. Fan and R. Li. “Variable Selection via Nonconcave Penalized Likelihood and Its Oracle Properties”. In: *Journal of the American statistical Association* 96.456 (2001), pp. 1348–1360.

[15] J. Weston et al. “Use of the Zero Norm with Linear Models and Kernel Methods”. In: *The Journal of Machine Learning Research* 3 (2003), pp. 1439–1461.

[16] W. J. Fu. “Penalized Regressions: The Bridge versus the Lasso”. In: *Journal of computational and graphical statistics* 7.3 (1998), pp. 397–416.

[17] B. D. Rao and K. Kreutz-Delgado. “An Affine Scaling Methodology for Best Basis Selection”. In: *Signal Processing, IEEE Transactions on* 47.1 (1999), pp. 187–200.

[18] D. Peleg and R. Meir. “A Bilinear Formulation for Vector Sparsity Optimization”. In: *Signal Processing* 88.2 (Feb. 2008), pp. 375–389.

[19] L. Heirendt et al. “Creation and Analysis of Biochemical Constraint-Based Models Using the COBRA Toolbox v.3.0”. In: *Nature Protocols* 14.3 (Mar. 2019), p. 639.

[20] A. K. Chavali et al. “Systems Analysis of Metabolism in the Pathogenic Trypanosomatid Leishmania Major”. In: *Molecular Systems Biology* 4 (Mar. 2008), p. 177.

[21] J. Förster et al. “Genome-Scale Reconstruction of the Saccharomyces Cerevisiae Metabolic Network”. In: *Genome Research* 13.2 (Feb. 2003), pp. 244–253.

[22] L. Caspeta et al. “Genome-Scale Metabolic Reconstructions of Pichia Stipitis and Pichia Pastoris and in Silico Evaluation of Their Potentials”. In: *BMC Systems Biology* 6 (2012), p. 24.

[23] R. Saha, P. F. Suthers and C. D. Maranas. “Zea Mays iRS1563: A Comprehensive Genome-Scale Metabolic Reconstruction of Maize Metabolism”. In: *PLoS ONE* 6.7 (July 2011).

[24] E. Brunk et al. “Recon3D Enables a Three-Dimensional View of Gene Variation in Human Metabolism”. In: *Nature Biotechnology* 36 (Feb. 2018), p. 272.

[25] J. L. Robinson et al. “An Atlas of Human Metabolism”. In: *Science Signaling* 13.624 (Mar. 2020).

[26] I. Thiele et al. “Personalized Whole-Body Models Integrate Metabolism, Physiology, and the Gut Microbiome”. In: *Molecular Systems Biology* 16.e8982 (2020), p. 24.

[27] J. Schellenberger et al. “Quantitative Prediction of Cellular Metabolism with Constraint-Based Models: The COBRA Toolbox v2.0”. In: *Nature Protocols* 6.9 (Sept. 2011), pp. 1290–1307.

[28] H.-G. Holzhütter. “The Principle of Flux Minimization and Its Application to Estimate Stationary Fluxes in Metabolic Networks.” In: *European journal of biochemistry / FEBS* 271.14 (2004), pp. 2905–22.

[29] I. Borodina, P. Krabben and J. Nielsen. “Genome-Scale Analysis of Streptomyces Coelicolor A3(2) Metabolism”. In: *Genome Research* 15.6 (Jan. 2005), pp. 820–829.

[30] J. Nogales, B. Ø. Palsson and I. Thiele. “A Genome-Scale Metabolic Reconstruction of Pseudomonas Putida KT2440: iJN746 as a Cell Factory.” In: *BMC Systems Biology* 2 (2008), p. 79.

[31] N. C. Duarte, M. J. Herrgård and B. Ø. Palsson. “Reconstruction and Validation of Saccharomyces Cerevisiae iND750, a Fully Compartmentalized Genome-Scale Metabolic Model”. In: *Genome Research* 14.7 (Jan. 2004), pp. 1298–1309.

[32] S. Dash et al. “Capturing the Response of Clostridium Acetobutylicumto Chemical Stressors Using a Regulated Genome-Scale Metabolic Model”. In: *Biotechnology for Biofuels* 7 (2014), p. 144.

[33] M. L. Mo, B. Ø. Palsson and M. J. Herrgård. “Connecting Extracellular Metabolomic Measurements to Intracellular Flux States in Yeast”. In: *BMC Systems Biology* 3.1 (Mar. 2009), p. 37.
